# Cardiorespiratory Fitness and Physical Activity in Pediatric Diabetes: A Systemic Review and Meta-Analysis

**DOI:** 10.1001/jamanetworkopen.2024.0235

**Published:** 2024-02-23

**Authors:** Hannah Steiman De Visser, Isaak Fast, Nicole Brunton, Edward Arevalo, Nicole Askin, Rasheda Rabbani, Ahmed M. Abou-Setta, Jonathan McGavock

**Affiliations:** 1 Children’s Hospital Research Institute of Manitoba, Winnipeg, Manitoba, Canada; 2 Neil John MacLean Library, Rady Faculty of Health Sciences, University of Manitoba, Winnipeg, Manitoba, Canada; 3 George & Fay Yee Centre for Healthcare Innovation, University of Manitoba, Winnipeg, Manitoba, Canada; 4 Department of Pediatrics and Child Health, University of Manitoba, Winnipeg, Manitoba, Canada; 5 Diabetes Research Envisioned and Accomplished in Manitoba (DREAM) Research Theme, Winnipeg, Manitoba, Canada; 6 Diabetes Action Canada, Toronto, Ontario, Canada

## Abstract

**Importance:**

It is unclear whether cardiorespiratory fitness (CRF) and physical activity are lower among youths with type 1 diabetes (T1D) and type 2 diabetes (T2D) compared with youths without diabetes.

**Objective:**

To describe the magnitude, precision, and constancy of the differences in CRF and physical activity among youths with and without diabetes.

**Data Sources:**

MEDLINE, Embase, CINAHL, and SPORTDiscus were searched from January 1, 2000, to May 1, 2022, for eligible studies.

**Study Selection:**

Observational studies with measures of CRF and physical activity in children and adolescents aged 18 years or younger with T1D or T2D and a control group were included.

**Data Extraction and Synthesis:**

Data extraction was completed by 2 independent reviewers. A random-effects meta-analysis model was used to estimate differences in main outcomes. The pooled effect estimate was measured as standardized mean differences (SMDs) with 95% CIs. The Preferred Reporting Items for Systematic Review and Meta-Analyses guideline was followed.

**Main Outcomes and Measures:**

The main outcomes were objectively measured CRF obtained from a graded maximal exercise test and subjective or objective measures of physical activity. Subgroup analyses were performed for weight status and measurement type for outcome measures.

**Results:**

Of 7857 unique citations retrieved, 9 studies (755 participants) with measures of CRF and 9 studies (1233 participants) with measures of physical activity for youths with T2D were included; for youths with T1D, 23 studies with measures of CRF (2082 participants) and 36 studies with measures of PA (12 196 participants) were included. Random-effects models revealed that directly measured CRF was lower in youths with T2D (SMD, −1.06; 95% CI, −1.57 to −0.56; *I*^2^ = 84%; 9 studies; 755 participants) and in youths with T1D (SMD, −0.39; 95% CI, −0.70 to −0.09; *I*^2^ = 89%; 22 studies; 2082 participants) compared with controls. Random-effects models revealed that daily physical activity was marginally lower in youths with T1D (SMD, −0.29; 95% CI, −0.46 to −0.11; *I*^2^ = 89%; 31 studies; 12 196 participants) but not different among youths with T2D (SMD, −0.56; 95% CI, −1.28 to 0.16; *I*^2^ = 91%; 9 studies; 1233 participants) compared with controls. When analyses were restricted to studies with objective measures, physical activity was significantly lower in youths with T2D (SMD, −0.71; 95% CI, −1.36 to −0.05; *I*^2^ = 23%; 3 studies; 332 participants) and T1D (SMD, −0.67; 95% CI, −1.17 to −0.17; *I*^2^ = 93%; 12 studies; 1357 participants) compared with controls.

**Conclusions and Relevance:**

These findings suggest that deficits in CRF may be larger and more consistent in youths with T2D compared with youths with T1D, suggesting an increased risk for cardiovascular disease–related morbidity in adolescents with diabetes, particularly among those with T2D. The findings reinforce calls for novel interventions to empower youths living with diabetes to engage in regular physical activity and increase their CRF.

## Key Points

**Question:**

Are cardiorespiratory fitness (CRF) and physical activity lower among youths with diabetes compared with youths without diabetes?

**Findings:**

In this systematic review and meta-analysis of 18 studies including 1988 youths with type 2 diabetes (T2D) and 59 studies with 14 278 youths with type 1 diabetes (T1D), CRF was 44.5% lower and physical activity 8.3% lower in youths with T2D compared with peers without diabetes. Both CRF and physical activity were 10% lower in youths with T1D compared with peers without diabetes.

**Meaning:**

These findings suggest that deficits in CRF are larger and more consistent in youths with T2D vs T1D, reinforcing calls for novel interventions to empower these youths to engage in regular physical activity.

## Teaser

This systematic review and meta-analysis evaluates cardiorespiratory fitness and physical activity differences between youths with diabetes and without diabetes.

## Introduction

Cardiorespiratory fitness (CRF)^1,2^ and physical activity^3,4^ in adolescence are important modifiable determinants of pediatric cardiometabolic disease and long-term mortality.^5^ Adolescents with high CRF^6,7^ and high daily physical activity^3,4^ are characterized as having lower blood pressure and lower central and overall obesity and may be less likely to develop cardiovascular disease–related morbidity in adulthood. Accordingly, engaging in regular physical activity^8,9^ and increasing CRF^10^ are consistent recommendations for optimal growth and health for children and adolescents, including those living with diabetes.^11,12,13,14,15^

Current clinical practice and expert guidelines argue that children and adolescents (referred to herein as youths) with type 1 diabetes (T1D) and type 2 diabetes (T2D) may be less active and have lower CRF than their peers without diabetes.^11,12,13,14,15^ Despite these claims, the evidence is mixed. Some studies have documented large deficits in physical activity and CRF in youth with T2D,^16^ while others have suggested that the differences are less profound.^17^ Among youths with T1D, some studies have found lower levels of physical activity and CRF,^18^ while many have suggested no difference^19^ or, in some cases, higher levels of physical activity.^20^ In the absence of a systematic review and meta-analysis of observational studies, the consistency, magnitude, and precision of these differences remain unclear. More accurate estimates of the differences in physical activity and CRF among youths living with chronic diseases is a priority among researchers in this area,^21^ and understanding the magnitude of these differences could help to tailor behavioral interventions to improve health outcomes for youths living with diabetes. To address these knowledge gaps in clinical pediatric diabetes, a systematic review and meta-analysis was done to determine whether (1) CRF and physical activity are lower among youths with diabetes compared with youths without diabetes and (2) to assess the overall quality of studies that describe differences in CRF and physical activity among youths with diabetes and their peers without diabetes.

## Methods

### Data Sources and Search Strategy

This systematic review and meta-analysis was conducted according to the Methodological Expectations of Cochrane Reviews^22^ and reported according to the Preferred Reporting Items for Systematic Reviews and Meta-Analyses (PRISMA) guideline.^23^ Institutional ethics approval was not required for this study as individual-level data were not used for the analysis.

We searched MEDLINE, Embase, CINAHL, and SPORTDiscus. Controlled vocabulary and free-text terms were used. A search for cross-sectional and cohort studies published between January 1, 2000, and May 1, 2022, was conducted (eTable 1 in Supplement 1). The systematic review followed a priori eligibility criteria, and the protocol was registered in the PROSPERO International Prospective Register of Systematic Reviews (CRD42022329303).

### Inclusion Criteria and Classification of Diabetes Type

We included cross-sectional and cohort studies that compared measures of CRF or physical activity between youths with T1D or T2D and controls without diabetes. The inclusion criteria for data extraction and meta-analyses were studies of youths up to age 18 years, cases of youths with T1D or T2D, a comparison group without diabetes, and published estimates of differences and variance for physical activity or CRF between youths with diabetes and controls without diabetes. We excluded experimental studies and studies that included data from adults (aged >18 years), compared youths with T1D and T2D directly without a comparison group without diabetes, or were published in languages other than English. We applied definitions established by Diabetes Canada^24,25^ and the American Diabetes Association^26,27^ to define T2D (hyperglycemia in the absence of markers of autoimmune disease) and T1D (autoimmunity and requirements for exogenous insulin) in youth.

### Study Selection

Abstracts and titles of relevant citations were independently screened by 3 reviewers (H.S.D., I.F., and E.A.) to determine eligibility. Prior to the full screen, a preliminary screen of 30 titles and abstracts was conducted to determine a preliminary agreement rating. As the preliminary agreement rate was adequate (28 of 30 abstracts were in agreement), the same reviewers independently screened all remaining titles and abstracts. Two reviewers (H.S.D. and I.F.) independently assessed the eligibility of full-text articles using a standardized prepiloted form outlining the inclusion and exclusion criteria. Disagreements were resolved by consensus or with the involvement of a third reviewer (J.M.).

### Data Extraction

Data were extracted independently by 2 reviewers (H.S.D. and I.F.), with disagreements resolved by consensus or with the involvement of a third reviewer (J.M.). Two authors (H.S.D. and I.F.) extracted data for study population demographics (age, sex, weight status, race and ethnicity [Black, Hispanic, Indigenous, White, and other], duration of diabetes, socioeconomic status) and outcome measures. We examined data on race and ethnicity because rates of T2D are disproportionally higher in racialized youths. We also created binary variables for the methods used to collect physical activity (objective vs subjective) and CRF (field vs laboratory) and within the measure of CRF, whether the tests were maximal or submaximal. Objective methods included pedometer- or accelerometer-based quantification of daily physical activity, while subjective methods included a range of self-reported tools. Laboratory-based methods to quantify CRF included graded maximal tests to exhaustion and submaximal tests with measured rates of oxygen consumption. Field tests of CRF included graded maximal tests to exhaustion (ie, shuttle run tests) that reported estimated rates of peak oxygen consumption (V̇o_2peak_) or time to exhaustion.

### Outcomes of Interest and Subgroup Analyses

Cardiorespiratory fitness was reported as the rate of maximal rate of oxygen consumption relative to body weight (milliliters per kilogram per minute) or mass of fat-free mass (milliliters per kilogram fat-free mass per min) or time to exhaustion at the end of a graded maximal exercise test. Physical activity was reported as minutes of physical activity per day, minutes of moderate to vigorous physical activity per day, metabolic equivalent tasks (METs), and MET-minutes and MET-hours per week. As there was substantial heterogeneity for the methods and reporting of both CRF and physical activity, we used the J-correction factor for an unbiased estimate of the standard mean difference (SMD) between youths with and without diabetes.^28^

### Risk-of-Bias Assessment

We evaluated the internal validity of included studies using a modified version of a risk-of-bias tool used in a previous meta-analysis of cross-sectional studies of physical activity for youths living with chronic diseases.^19^ This risk-of-bias tool consists of several domains specific to measurements of physical activity and CRF, including type of measurement (objective vs subjective), time of year for data collection, data reduction, criteria used to define a valid measurement, and reporting of confounding. Each domain was judged as yes or no and given a score of 1 or 0 for the presence or absence of each potential source of bias. The overall risk-of-bias score was based on the responses to individual domains.

### Statistical Analysis

The primary outcomes for the meta-analyses were SMDs in physical activity and CRF. When at least 2 studies of similar populations, methods, and outcomes were available, inverse variance–weighted random-effects meta-analyses were performed to calculate SMDs with 95% CIs between youths with and without diabetes. We initially calculated SMDs with 95% CIs for all studies that met the inclusion criteria. Absolute mean differences with 95% CIs were calculated for differences in the main outcomes for subgroup analyses of studies that used identical tools to quantify outcome measures between youths with and without diabetes. The range of differences and the inconsistency index (*I*^2^) were both used to quantify heterogeneity among studies.^28,29^ Subgroup analyses were performed to determine if differences in outcomes were influenced by (1) the methods used to assess the main outcomes of interest (direct vs indirect measures for CRF and subjective vs objective measures for physical activity) and (2) the type of control population studied (healthy weight vs weight matched). We also performed sensitivity analyses by removing extreme outliers. Funnel plots were used to investigate publication bias using the Egger test and visual inspection to assess plot asymmetry for all 4 meta-analyses. The meta-analyses were conducted using the general meta and metafor packages^30^ in RStudio, version 2022.07.2 + 576 and R, version 4.2.2 (R Project for Statistical Computing).

## Results

The search results yielded 7857 unique individual citations, which were included in the preliminary screen. At the end of the title and abstract screen, 7400 articles were excluded, 52 were included, and 405 were in conflict across the 3 reviewers. There was a 94.8% agreement rate among the reviewers for the title and abstract screen. The 405 articles with conflicting scores were resolved by the 3 reviewers and 1 author (J.M.) to determine consensus for eligibility for full-article screening and data extraction. After all the conflicts were resolved, 130 articles were included for the full-article screen, and 54 studies were included for data extraction (eFigure 1 in Supplement 1).^16,17,18,20,31,32,33,34,35,36,37,38,39,40,41,42,43,44,45,46,47,48,49,50,51,52,53,54,55,56,57,58,59,60,61,62,63,64,65,66,67,68,69,70,71,72,73,74,75,76,77,78,79,80^ Details for each individual study are presented in eTables 2 to 5 in Supplement 1. For all 4 meta-analyses, cases and controls were similar in age and sex (Table 1), with limited data available for race and ethnicity across all studies (eTables 6 and 7 in Supplement 1).

**Table 1.** Studies Included in the Systematic Review and Meta-Analysis

	T2D and CRF^a^		T1D and CRF^b^		T2D and PA^c^		T1D and PA^d^	
	Cases	Controls	Cases	Controls	Cases	Controls	Cases	Controls
No. of participants	286	469	1018	1064	442	791	3209	8993
Age, median (range), y	15.1 (12.9-16.4)	15.1 (12.5-16.2)	14.1 (10.5-16.2)	13.5 (10.1-16.0)	15.2 (12.9-16.4)	14.8 (12.5-16.0)	14.0 (7.4-16.0)	13.6 (7.3-16.3)
BMI, median (range)	34.3 (25.3-42.5)	26.6 (20.3-35)	21.3 (18.0-31.1)	21.43 (17-32.9)	31.9 (25.3-35.0)	24.9 (21.0-32.3)	20.78 (16.6-24.6)	20.32 (17.0-32.9)
Sex, No. (%)								
Girls	161 (56.2)	277 (59.1)	465 (45.7)	529 (49.7)	301 (68)	464 (59)	1653 (51.6)	4559 (50.7)
Boys	125 (43.8)	192 (40.9)	553 (54.3)	535 (50.3)	141 (32)	327 (41)	1550 (48.4)	4434 (49.3)
V̇o_2peak_, mean (SD), mL/kg/min	20.6 (3.5)	25.1 (5.4)	36.9 (5.3)	40.2 (5.9)	NA	NA	NA	NA
MVPA, median (range), min/d	NA	NA	NA	NA	30.4^e^	53.4^e^	54.6 (42.4-82.9)	73.7 (53.4-114.0)

Abbreviations: BMI, body mass index (weight in kilograms divided by height in meters squared); CRF, cardiorespiratory fitness; MVPA, moderate to vigorous physical activity; NA, not applicable; PA, physical activity; T1D, type 1 diabetes; T2D, type 2 diabetes; V̇o_2peak_, peak oxygen consumption.

^a^ Nine studies.^16,17,35,44,46,49,61,72,79^

^b^ Twenty-two studies.^18,34,38,39,40,44,45,46,48,50,51,53,56,58,65,67,69,72,73,75,77,78^

^c^ Nine studies.^16,17,35,44,55,61,66,71,76^

^d^ Thirty-one studies.^18,33,34,36,37,39,40,41,42,43,44,47,48,52,54,55,57,58,59,60,64,65,66,70,73,74,75,78^

^e^ MVPA reported in minutes per day in only 1 study.^44^

**Figure 1.**
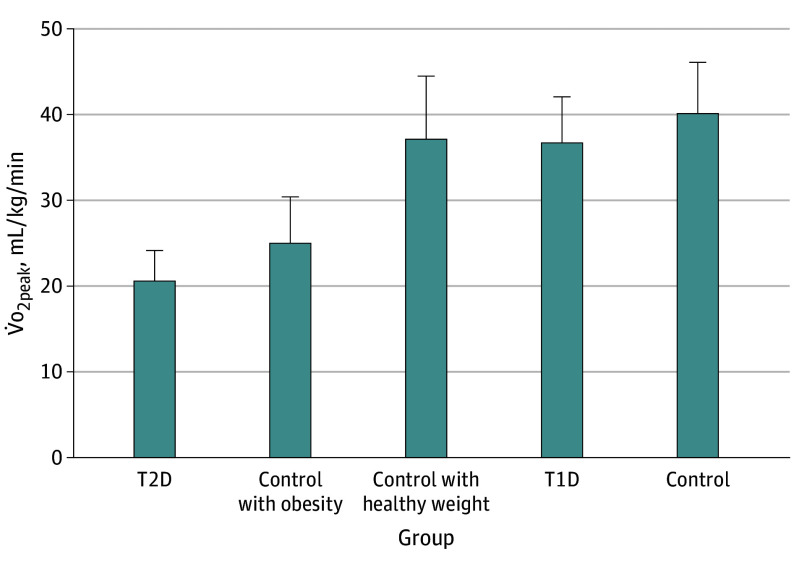
Differences in Peak Oxygen Consumption (V̇o_2peak_) Between Youths Without Diabetes and Youths With Type 1 Diabetes (T1D) and Type 2 Diabetes (T2D) The bars indicate the means; whiskers indicate SDs.

**Figure 2.**
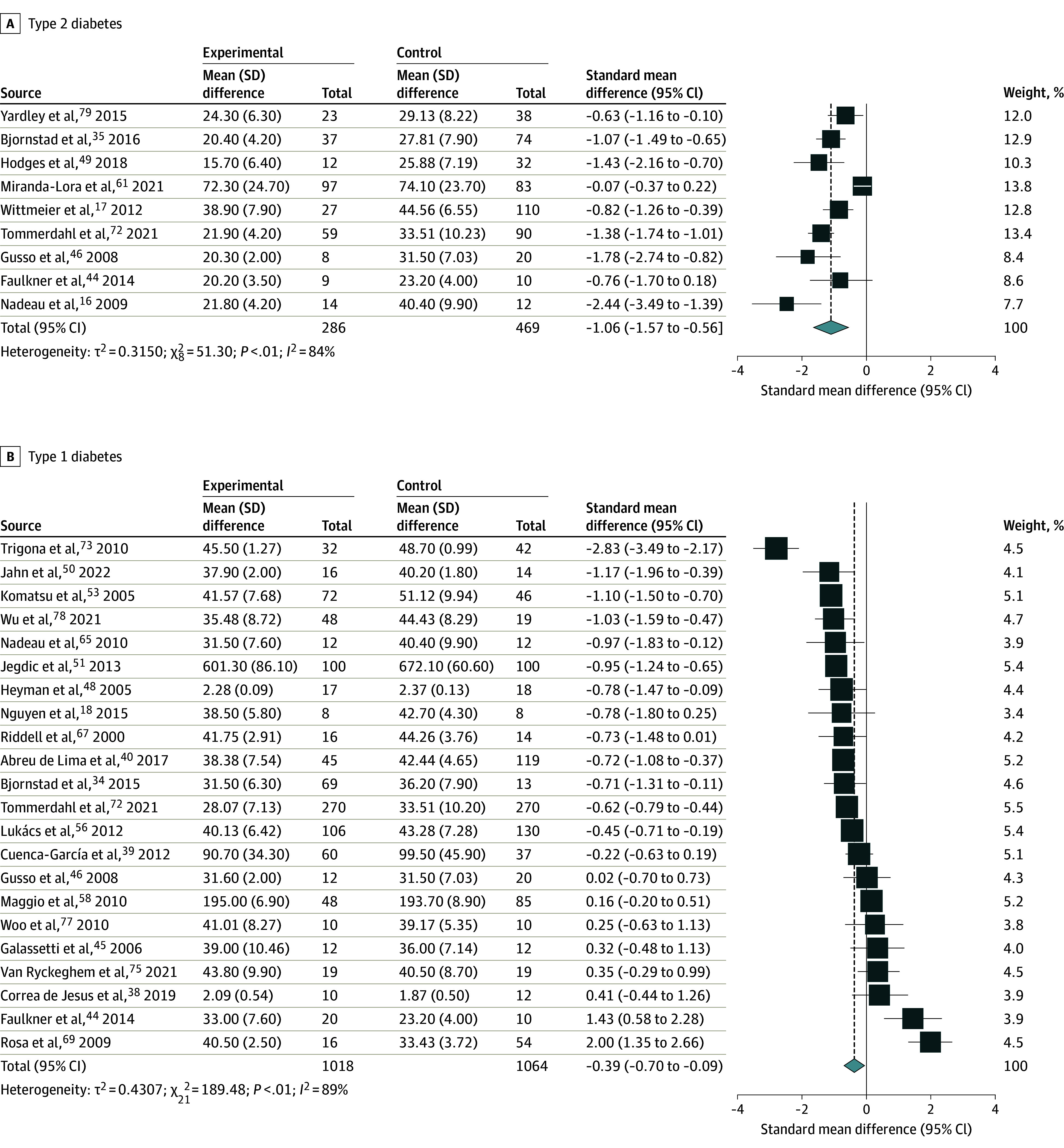
Differences in Cardiorespiratory Fitness Between Youths With Diabetes and Controls The boxes and whiskers indicate standardized mean differences and 95% CIs.

Among the 55 studies included in data extraction, 9 studies (755 participants, including 286 cases [median (range) age, 15.1 (12.9-16.4) years; 161 girls (56.2%); 125 boys (43.8%)] and 469 controls [median (range) age, 15.1 (12.5-16.2) years; 277 girls (59.1%); 192 boys (40.9%)]) met the eligibility criteria for the outcome of CRF for youths with T2D,^16,17,35,44,46,49,61,72,79^ and 22 studies (38 unique comparisons; 2082 participants; 1018 cases [median (range) age, 14.1 (10.5-16.2) years; 465 girls (45.7%); 553 boys (54.3%)] and 1064 controls [median (range) age, 13.5 (10.1-16.0) years; 529 girls (49.7%); 535 boys (50.3%)]) met the eligibility criteria for the outcome of CRF for youths with T1D^18,34,38,39,40,44,45,46,48,50,51,53,56,58,65,67,69,72,73,75,77,78^ (Table 1). Mean (SD) V̇o_2peak_ values from laboratory-based studies were 20.7 (3.5) mL/kg/min for youths with T2D (44.6% lower than controls with healthy weight and without diabetes and 17.4% for weight-matched controls) and 36.9 (5.3) mL/kg/min for youths with T1D (8.3% lower than controls without diabetes) (Figure 1). The random-effects meta-analysis revealed that youths with T2D displayed significantly lower CRF compared with controls without diabetes (SMD, −1.06; 95% CI, −1.57 to −0.56; *I*^2^ = 84%; 9 studies^16,17,35,44,46,49,61,72,79^; 755 participants; 286 cases) (Figure 2A). When data were restricted to studies with laboratory-based measures of V̇o_2peak_, youths with T2D displayed significantly lower CRF compared with controls without diabetes (mean difference, −8.76 mL/kg/min; 95% CI, −12.7 to −4.9 mL/kg/min; *I*^2^ = 82%; 8 studies^16,17,35,44,46,49,72,79^; 575 participants).

The random-effects meta-analysis revealed that CRF was also lower in youths with T1D compared with controls without diabetes (SMD, −0.39; 95% CI, −0.70 to −0.09; *I*^2^ = 89%; 22 studies^18,34,38,39,40,44,45,46,48,50,51,53,56,58,65,67,69,72,73,75,77,78^; 2082 participants; 1018 cases) (Figure 2B). Restricting the analysis to laboratory-based measures of CRF did not change the estimate of the difference between youths with T1D and controls without diabetes (mean difference, −3.9 mL/kg/min; 95% CI, −6.3 to −1.6 mL/kg/min; *I*^2^ = 68%; 13 studies^18,34,40,45,50,53,56,65,67,73,75,77,78^; 917 participants). Subgroup analyses of studies of youths with T2D revealed that the differences in CRF were lower for controls without diabetes who had obesity (SMD, −0.75; 95% CI, −1.15 to −0.35; *I*^2^ = 77%; 8 studies^17,35,44,46,49,61,72,79^; 593 participants) compared with studies with controls who had a healthy weight (SMD, −2.10; 95% CI, −2.74 to −1.47; *I*^2^ = 77%; 7 studies^16,17,35,46,49,72,79^; 328 participants). Subgroup analyses restricted to youths with T1D with a healthy weight revealed that CRF was lower compared with controls with a healthy weight and without diabetes (SMD, −0.75; 95% CI, −1.09 to −0.41; *I*^2^ = 86%; 15 studies^18,34,39,48,50,51,53,56,58,65,72,73,75,77,78^; 1295 participants). Only 2 studies^46,72^ performed in youths with T1D who had obesity showed no differences in CRF compared with controls without diabetes.

**Figure 3.**
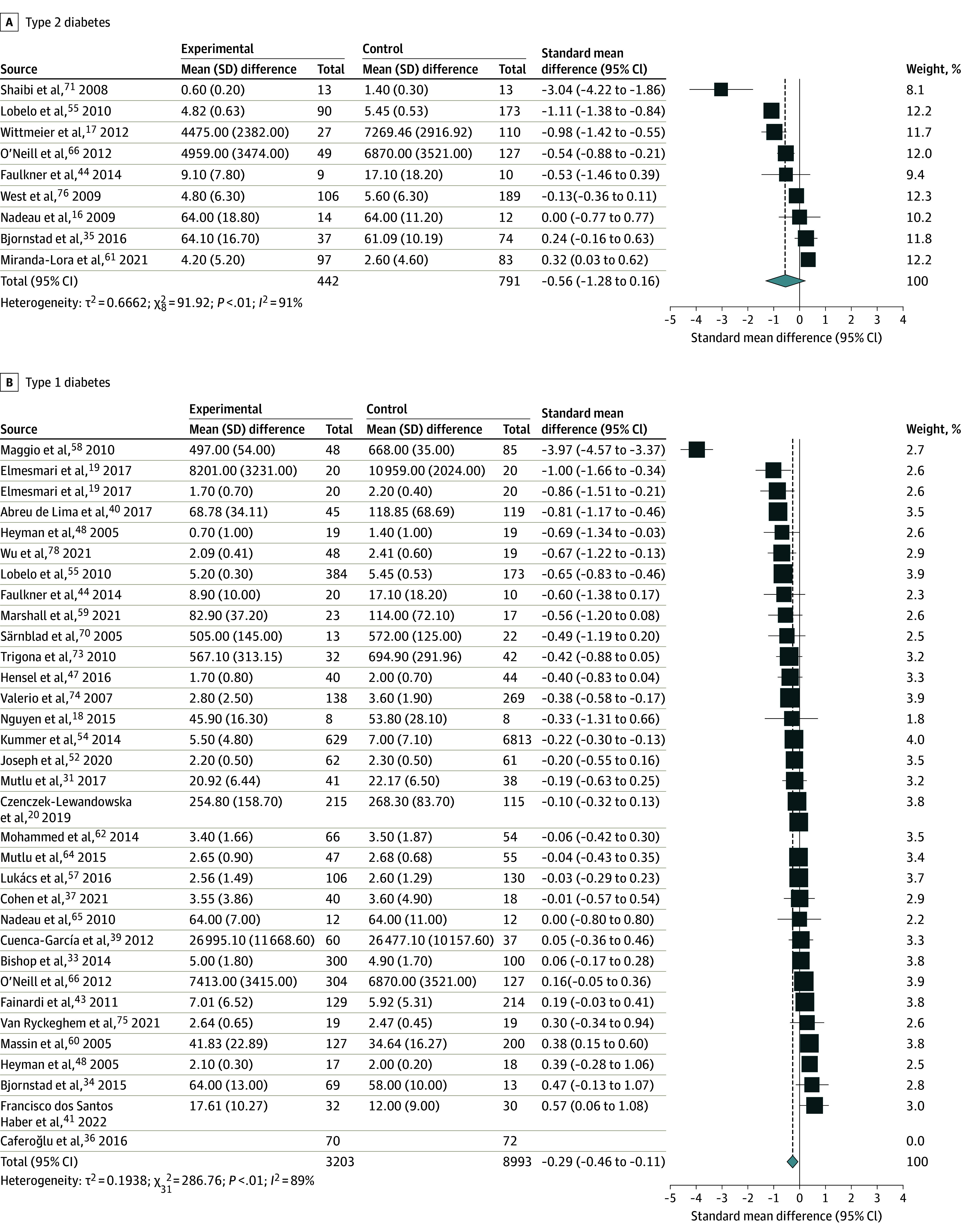
Differences in Daily Physical Activity Between Youths With Diabetes and Controls Heyman et al^48^ had 2 control groups and was entered twice in the meta-analysis. Elmesmari et al^42^ used 2 different measures for the same study. The boxes and whiskers indicate standardized mean differences and 95% CIs.

Of the 55 studies that met eligibility for data extraction, 9 studies (10 unique comparisons; 1233 participants, including 442 cases [median (range) age, 15.2 (12.9-16.4) years; 301 girls (68%); 141 boys (32%)] and 791 controls [median (range) age, 14.8 (12.5-16.0) years; 464 girls (59%); 327 boys (41%)]) met eligibility for the outcome of physical activity for youths with T2D,^16,17,35,44,55,61,66,71,76^ and 31 studies (12 196 participants, including 3203 cases [median (range) age, 14.0 (7.4-16.0) years; 1653 girls (51.6%); 1550 boys (48.4%)] and 8993 controls [median (range) age, 13.6 (7.3-16.3) years; 4559 girls (50.7%); 4434 boys (49.3%)]) met eligibility for the outcome of physical activity for youths with T1D^18,19,20,33,34,36,37,39,40,41,42,43,44,47,48,52,54,55,57,58,59,60,62,64,65,66,70,73,74,75,78^ (Table 1). Random-effects models revealed that daily physical activity was not different among youths with T2D (SMD, −0.56; 95% CI, –1.28 to 0.16; *I*^2^ = 91%; 9 studies^16,17,35,44,55,61,66,71,76^; 1233 participants; 442 cases) (Figure 3A) compared with controls without diabetes but was marginally lower among youths with T1D (SMD, −0.29; 95% CI, –0.46 to −0.11; *I*^2^ = 89%; 31 studies^18,19,20,33,34,36,37,39,40,41,42,43,44,47,48,52,54,55,57,58,59,60,62,64,65,66,70,73,74,75,78^; 12 196 participants; 3203 cases) (Figure 3B) compared with controls without diabetes. Analyses restricted to studies with objective measures found that physical activity was lower in youths with T2D (SMD, −0.71; 95% CI, −1.36 to −0.05; *I*^2^ = 23%; 3 studies^17,44,66^; 332 participants) and T1D (SMD, −0.67; 95% CI, −1.17 to −0.17; *I*^2^ = 93%; 13 studies^18,20,39,42,44,58,59,65,66,70,73,78^ [Elmesmari et al^42^ used 2 different measures for the same study]; 1357 participants) compared with controls without diabetes (eFigures 2 and 3 in Supplement 1).

To determine the overall quality of the studies in this area, we summarized the distribution of bias for studies of CRF and physical activity among youths with T2D and T1D (Table 2). For all studies combined, 78% to 89% of studies provided information on the recruitment process for the comparison population. Only 22% to 36% of studies described the timing of the measurements of physical activity, 22% to 39% of all studies described methods to address potential sources of bias, 14% to 44% adjusted for confounding, and 0% to 22% presented sex-disaggregated data. For studies of youths with T2D, the majority (8 of 9 studies^17,35,44,46,49,61,72,79^) relied on laboratory-based measures of CRF. For studies of physical activity among youths with T2D, only 3 of 9 studies^17,44,66^ used objective measures, and none of the studies provided definitions for a valid assessment of physical activity or described the season during which data were collected. For studies of CRF among youths with T1D, the majority relied on laboratory-based maximal tests to exhaustion (21 of 23 studies^18,34,38,39,40,44,45,46,48,50,53,58,65,67,68,69,72,73,77,78^), but only some reported criteria for achieving maximal exertion.^18,34,39,40,44,45,48,53,58,68,72,73,77,78^ For studies of physical activity among youths with T1D, 13 of 31 used objective measures^18,20,39,42,44,58,59,65,66,70,73,78^ (Elmesmari et al^42^ used 2 different measures for the same study) and 11 of 13 studies provided definitions for a valid assessment of physical activity.^18,20,39,42,44,58,59,65,70,73^ Funnel plots revealed no publication bias for the 4 meta-analyses presented above (eFigures 4-7 in Supplement 1).

**Table 2.** Summary of Risk Scores Across Different Populations and Outcomes

Variable	No. of studies (%)			
	T1D		T2D	
	PA (n = 31)	CRF (n = 22)	PA (n = 9)	CRF (n = 9)
Objective/direct	14 (45.2)	20 (90.9)	4 (44.4)	8 (88.9)
Sample recruitment	27 (87.1)	18 (81.8)	8 (88.9)	7 (77.8)
Time of year	11 (35.5)	1 (4.5)	2 (22.2)	0
Place of recruitment	27 (87.1)	16 (72.7)	7 (77.8)	7 (77.8)
Sample description, mean (maximum score = 3)	2.84	2.77	2.89	2.90
Attrition, mean (maximum score = 2)	1.41	1.41	1.44	1.5
Data collection and reduction, mean (maximum score = 5)	2.63	3.72	1.44	3.20
PA intensity/maximum criteria definition	19 (61.2)	16 (72.7)	3 (33.3)	5 (55.6)
Results (No. actually analyzed)	31 (100)	22 (100)	8 (88.9)	9 (100)
Control recruitment described	23 (74.1)	15 (68.2)	8 (88.9)	7 (77.8)
Eligibility criteria described	20 (64.5)	19 (86.4)	5 (55.6)	8 (88.9)
Address bias	11 (35.5)	8 (36.4)	3 (33.3)	2 (22.2)
Report confounding	19 (61.2)	17 (77.2)	8 (88.9)	8 (88.9)
Adjust for confounding	4 (12.9)	4 (18.2)	3 (33.3)	4 (44.4)
Sex-disaggregated data	5 (16.1)	5 (22.7)	0	1 (11.1)
Ethnicity data, mean (maximum score = 3)	0.41	0.18	1.33	0.70
Total score, mean (maximum score = 25)	13.75	15.40	13.67	15.20

Abbreviations: CRF, cardiorespiratory fitness; PA, physical activity; T1D, type 1 diabetes; T2D, type 2 diabetes.

## Discussion

The main finding from this systematic review and meta-analysis was that youths with T2D had very low CRF, with values 17% to 45% lower than in age-matched controls without diabetes, and modestly lower daily physical activity. The mean V̇o_2peak_ for youths with T2D (20.7 mL/kg/min) was well below normative values for adolescents and established thresholds for optimal cardiometabolic risk profiles.^2,81,82,83^ Among youths with T1D, both CRF and physical activity were modestly lower compared with age-matched controls without diabetes. The differences in physical activity between adolescents with diabetes and controls were more evident when objective measures were used to quantify physical activity. Finally, most studies were poorly designed and did not follow current reporting guidelines, which may explain the high heterogeneity of effect sizes across studies.

Low CRF was associated with increased cardiometabolic risk factor clustering in adolescence and cardiovascular disease–related morbidity in adulthood.^2,10,84,85^ The mean V̇o_2peak_ derived from direct laboratory-based measures for youths with T2D was 20.7 mL/kg/min, a value approximately 45% lower than in controls with a healthy weight and without diabetes. This deficit was largely mediated by excess body weight, as differences with weight-matched controls were approximately 17%. In contrast, adolescents with T1D had a mean V̇o_2peak_ of 36.9 mL/kg/min, a value approximately 10% lower than controls without diabetes. These levels of CRF for youth with T2D are well below mean values for representative samples of adolescents in the US,^81^ Canada,^82^ and Europe^83^ and below the minimum threshold at which the odds of cardiometabolic risk factor clustering increase 2- to 4-fold (<41.6 mL/kg/min for boys; <34.6 mL/kg/min for girls).^2^ These deficits in CRF in youths living with diabetes may therefore contribute partially to the cardiovascular disease risk factor clustering reported in adolescence^31^ and excessive cardiovascular disease–related morbidity observed in young adulthood.^86,87^ These data also support clinical practice guidelines and expert opinions that CRF is impaired in youths with both T1D and T2D.^11,12,13,14,15^

Engaging in daily physical activity is a cornerstone in the management of pediatric diabetes.^11,12,13,14,15^ Despite the widely recognized benefits of physical activity, very few systematic reviews with meta-analyses have been published describing daily physical activity levels in youths living with diabetes. One meta-analysis of cross-sectional studies of adolescents with chronic diseases found no difference in moderate to vigorous physical activity among youths with T1D compared with a control population.^17^ With an additional 5 studies,^20,42,47,66,78^ we found a very small difference in daily physical activity among youths with T1D compared with peers without diabetes. Fewer studies have documented levels of daily physical activity among youths with T2D; however, they consistently revealed very low levels of daily physical activity compared with expert recommendations (approximately 7000 steps per day and approximately 35 minutes of moderate to vigorous physical activity daily).^17,88,89,90^ Compared with youths without diabetes, the differences in daily physical activity were greater for youths with T2D compared to youths with T1D. The large deficits in daily physical activity among youths with T2D may contribute to the lower CRF reported here, reinforcing the need for novel approaches to support physical activity behaviors for adolescents with T2D.

### Limitations

This systematic review is, to our knowledge, the largest and most comprehensive description of CRF and physical activity levels among youths living with diabetes to date. Although we applied strict criteria for study inclusion and published the methods a priori, there are several limitations to consider. First, the quality of a meta-analysis depends on the quality of studies included. One of the main objectives of this systematic review was to determine the quality of studies in this area. As the majority of studies did not report key methodological considerations for valid measures of physical activity and CRF, consistently describe strategies to address bias (including weight status), or present sex-specific results, the risk of bias was high in most studies. Therefore, the point estimates provided here should be interpreted with caution. Second, the external validity or transferability of the results is also limited by several factors, including failure to report race and ethnicity; socioeconomic status; and, in most cases, sex-specific differences in CRF and physical activity within each population. Structural factors that influence access, safety, and participation in physical activity are important when studying differences in physical activity for youths of various racial and ethnic identities, who are new immigrants, and who identify as female or nonbinary. Type 2 diabetes is more common among American Indian, Black, and Hispanic youths^91^ and those living in low-income households^92^; however, despite calls for reporting sex- and race and ethnicity–specific outcomes, these data are largely absent from studies published to date. Finally, we did not include non-English publications or studies within the gray literature in order to increase the feasibility of this review, and this may have introduced selective reporting bias (eg, publication bias).

## Conclusions

The findings of this systematic review and meta-analysis suggest that youths with T2D have profoundly lower CRF and modestly lower objectively measured physical activity compared with peers without diabetes. Deficits in CRF and objectively measured physical activity are also evident, but less pronounced, among youths with T1D. These findings reinforce calls for novel interventions to empower youths living with diabetes to engage in regular physical activity and increase their CRF.

## Notes

Supplement 1. **eTable 1.** Search Strategies and Results **eTable 2.** Baseline Characteristics of Included Studies for Youth With Type 1 Diabetes and Cardiorespiratory Fitness **eTable 3.** Baseline Characteristics of Included Studies for Youth With Type 1 Diabetes and Physical Activity **eTable 4.** Baseline Characteristics of Included Studies for Youth With Type 2 Diabetes and Physical Activity **eTable 5.** Baseline Characteristics of Included Studies for Youth With Type 2 Diabetes and Cardiorespiratory Fitness **eTable 6.** Ethnicity of Participants in Each Study of Youth With Type 2 Diabetes Within Studies That Reported It **eTable 7.** Ethnicity of Participants in Each Study of Youth With Type 1 Diabetes **eFigure 1.** Flow Chart Describing Search and Screening Results for Studies That Were Included in the Meta-Analysis **eFigure 2.** Differences in Objectively Measured Physical Activity Between Youth With Type 2 Diabetes and Controls **eFigure 3.** Differences in Objectively Measured Physical Activity Between Youth With Type 1 Diabetes and Controls **eFigure 4.** Funnel Plot Demonstrating No Publication Bias in Studies Examining Differences in Cardiorespiratory Fitness Between Youth With Type 2 Diabetes and Controls **eFigure 5.** Funnel Plot Demonstrating No Publication Bias in Studies Examining Differences in Cardiorespiratory Fitness Between Youth With Type 1 Diabetes and Controls **eFigure 6.** Funnel Plot Demonstrating No Publication Bias in Studies Examining Differences in Physical Activity Between Youth With Type 2 Diabetes and Controls **eFigure 7.** Funnel Plot Demonstrating No Publication Bias in Studies Examining Differences in Physical Activity Between Youth With Type 1 Diabetes and Controls

Supplement 2.
Data Sharing Statement
