# Supplementary material for: Cardiorespiratory Fitness and Physical Activity in Pediatric Diabetes: A Systemic Review and Meta-Analysis
Source: JAMA Netw Open. 2024 Feb 23;7(2):e240235. doi: 10.1001/jamanetworkopen.2024.0235 (PMC10891480; doi:10.1001/jamanetworkopen.2024.0235)
Supplement: Supplement 1. — eTable 1. Search Strategies and Results eTable 2. Baseline Characteristics of Included Studies for Youth With Type 1 Diabetes and Cardiorespiratory Fitness eTable 3. Baseline Characteristics of Included Studies for Youth With Type 1 Diabetes and Physical Activity eTable 4. Baseline Characteristics of Included Studies for Youth With Type 2 Diabetes and Physical Activity eTable 5. Baseline Characteristics of Included Studies for Youth With Type 2 Diabetes and Cardiorespiratory Fitness eTable 6. Ethnicity of Participants in Each Study of Youth With Type 2 Diabetes Within Studies That Reported It eTable 7. Ethnicity of Participants in Each Study of Youth With Type 1 Diabetes eFigure 1. Flow Chart Describing Search and Screening Results for Studies That Were Included in the Meta-Analysis eFigure 2. Differences in Objectively Measured Physical Activity Between Youth With Type 2 Diabetes and Controls eFigure 3. Differences in Objectively Measured Physical Activity Between Youth With Type 1 Diabetes and Controls eFigure 4. Funnel Plot Demonstrating No Publication Bias in Studies Examining Differences in Cardiorespiratory Fitness Between Youth With Type 2 Diabetes and Controls eFigure 5. Funnel Plot Demonstrating No Publication Bias in Studies Examining Differences in Cardiorespiratory Fitness Between Youth With Type 1 Diabetes and Controls eFigure 6. Funnel Plot Demonstrating No Publication Bias in Studies Examining Differences in Physical Activity Between Youth With Type 2 Diabetes and Controls eFigure 7. Funnel Plot Demonstrating No Publication Bias in Studies Examining Differences in Physical Activity Between Youth With Type 1 Diabetes and Controls [file jamanetwopen-e240235-s001.pdf]

Supplemental Online Content

Steiman De Visser H, Fast I, Brunton N, et al. Cardiorespiratory fitness and physical activity in pediatric diabetes: a systematic review and meta-analysis. *JAMA Netw Open*. 2024;7(2):e240235. doi:10.1001/jamanetworkopen.2024.0235

eTable 1. Search Strategies and Results

eTable 2. Baseline Characteristics of Included Studies for Youth With Type 1 Diabetes and Cardiorespiratory Fitness

eTable 3. Baseline Characteristics of Included Studies for Youth With Type 1 Diabetes and Physical Activity

eTable 4. Baseline Characteristics of Included Studies for Youth With Type 2 Diabetes and Physical Activity

eTable 5. Baseline Characteristics of Included Studies for Youth With Type 2 Diabetes and Cardiorespiratory Fitness

eTable 6. Ethnicity of Participants in Each Study of Youth With Type 2 Diabetes Within Studies That Reported It

eTable 7. Ethnicity of Participants in Each Study of Youth With Type 1 Diabetes Within Studies That Reported It

eFigure 1. Flow Chart Describing Search and Screening Results for Studies That Were Included in the Meta-Analysis

eFigure 2. Differences in Objectively Measured Physical Activity Between Youth With Type 2 Diabetes and Controls

eFigure 3. Differences in Objectively Measured Physical Activity Between Youth With Type 1 Diabetes and Controls

eFigure 4. Funnel Plot Demonstrating No Publication Bias in Studies Examining Differences in Cardiorespiratory Fitness Between Youth With Type 2 Diabetes and Controls

eFigure 5. Funnel Plot Demonstrating No Publication Bias in studies Examining Differences in Cardiorespiratory Fitness Between Youth With Type 1 Diabetes and Controls

eFigure 6. Funnel Plot Demonstrating No Publication Bias in Studies Examining Differences in Physical Activity Between Youth With Type 2 Diabetes and Controls

eFigure 7. Funnel Plot Demonstrating No Publication Bias in Studies Examining Differences in Physical Activity Between Youth With Type 1 Diabetes and Controls

This supplemental material has been provided by the authors to give readers additional information about their work.

© 2024 Steiman De Visser H et al. *JAMA Network Open.*

eTable 1. Search Strategies and Results

Medline

Search	Publications
adolescent/ or exp child/ or exp infant/ or minors/ or exp puberty/ or adolescent health services/ or exp child health services/ or adolescent medicine/ or exp pediatrics/ or adolescent health/ or infant health/ or child health/ or exp pediatricians/ or pediatric nurse practitioners/ or exp nurses, pediatric/ or exp pediatric nursing/ or pediatric assistants/ or hospitals, pediatric/	3884104
(adolesc* or babies or baby or boy? or boyhood or girlhood or child* or girl? or infan* or juvenil* or kid? or minor? or stepchild* or neonat* or neo-nat* or newborn* or new-born* or paediatric* or peadiatric* or pediatric* or pubert* or pubescen* or prepubert* or prepubescen* or preschool* or kindergarten* or school* or highschool* or youngster* or preteen* or teen* or toddler* or underage? or under-age? or youth*).ti,ab,kf	3123278
(pediatric* or paediatric* or infan* or child* or adolescen*).jn,jw	717227
(pediatric* or paediatric* or infan* or child* or adolescen*).in	1266163
or/1-4	5488579
Diabetes Mellitus/ or exp diabetes mellitus, type 1/ or exp diabetes mellitus, type 2/	349325
(diabet* or IDDM or NIDDM or T1DM or T2DM or T1D or T2D).ti,ab,kf	726244
or/6-7	764923
exp exercise/ or exp exercise movement techniques/ or exp exercise therapy/ or dancing/ or exp physical fitness/ or exp sports/ or aquatic therapy/ or exp exercise test/ or ergometry/ or exp accelerometry/ or physical exertion/ or exp physical endurance/ or exp oxygen consumption/ or fitness trackers/	537835
(exercis* or exertion or endurance or stamina or fitness* or sedentary or physiotherap* or physio therap* or kinesiotherap* or athletic* or workout or gym or gymnasi* or ymca* or work* out).ti,ab,kf	507220
((physical* or cardio* or aerobic*) adj4 (activ* or fit* or capacit* or effort* or inactiv* or conditioning or recondition* or train*)).ti,ab,kf	221279
((active or activit*) adj2 (living or play* or gaming or game* or lifestyle or behavio?r* or track* or monitor* or sensing or sensor* or detect*)).ti,ab,kf	90445

© 2024 Steiman De Visser H et al. *JAMA Network Open.*

(crossfit* or HIIT or HIIE or plyometric* or personal train* or bodybuild* or body build* or weightlift* or (weight* adj2 lift*)).ti,ab,kf	8686
((train* or condition* or program* or session*) adj4 (weight* or strength* or enduranc* or resistance or athletic* or fitness or aerobic* or interval* or flexibility or balance or muscle* or muscular or circuit or isometric* or isotonic* or agility or power)).ti,ab,kf	125292
(treadmill* or tread-mill* or walk* or stair* or jog* or swim* or running or runner* or run or runs or marathon* or triathl* or dance or dancing or climb* or cycling or bicycl* or bike or bikes or exercycl* or exerbik* or calisthenic* or skateboard* or rowing or rower or canoe* or kayak* or paddl* or skate or skating or ski or skiing or hike or hiking or jump or jumping or (skip* adj2 rope*)).ti,ab,kf	525796
(tai ji or tai chi or ai chi or yoga or pilates or stretch or stretches or stretching or flexibility or movement or qigong or qi gong or barre or tae bo or zumba or jazzercise).ti,ab,kf	435661
(pedomet* or accelerom* or actimet* or actigraph* or ergomet* or ergonomet* or navette? or shuttle or cycloergomet* or effort test* or andersen test* or maxim* oxygen* or peak oxygen* or oxygen consum* or oxygen require* or oxygen demand* or eurofit or prefit or pre-fit or president* challenge* or youth physical program* or physical best or stress test* or vo2max or vo2 max or an?erobic threshold* or metabolic equivalen* or work* rate* or work* capacit* or (time adj2 (exhaust* or fatigue*)) or ((step or steps) adj3 (count* or test* or daily or day or days or track*))).ti,ab,kf	175548
(exergam* or wii fit or zwift or peloton or kinect or fitbit*).ti,ab,kf	3492
(martial art* or boxing or kickboxing or karate or judo or jiu jitsu or jiujitsu or taekwondo or tae kwon do or fencing or wrestl* or aikido or fight* or grappl*).ti,ab,kf	48218
(sport* or golf* or aquatic* or gymnastic* or tennis or badminton or volleyball or ball or basketball or baseball or bowling or curls or curling or dodgeball or frisbee or lacrosse or racquet* or ringette or softball or polo or cricket or hockey or football or soccer or rugby or handball or mountaineer*).ti,ab,kf	214286
or/9-20	1978304
exp epidemiologic studies/	2954408
(case control? or case comparison? or case base? or case referent or case referrent or case compeer or matched case?).tw,kf.	153468
(cohort adj (study or studies or analy$)).tw,kf.	286833
control* adj2 (before-after or "before and after").tw,kf	3887

© 2024 Steiman De Visser H et al. *JAMA Network Open.*

historic* adj2 control*.tw,kf	9659
((Follow up or followup) adj (study or studies)).tw,kf.	56197
((observation* or epidemiologic* or comparative or comparison or correlation*) adj2 (study or studies)).tw,kf.	434262
(longitudinal or retrospective or prospective).tw,kf.	1528593
(Cross section* or crosssection*).tw,kf.	494340
comparative study.pt or observational study.pt	2027826
or/22-31	5386529
5 and 8 and 21 and 32	3926
exp animals/ not humans/	5009925
33 not 34	3893
limit 35 to (english language and yr="2000 -Current")	3280

© 2024 Steiman De Visser H et al. *JAMA Network Open.*

Embase

Search	Publications
exp adolescent/ or exp child/ or "minor (person)"/ or exp puberty/ or exp child health care/ or exp pediatrics/ or exp adolescent health/ or child health/ or exp pediatrician/ or neonatologist/ or pediatric nurse practitioner/ or pediatric nurse/ or neonatal nurse/ or exp pediatric nursing/ or pediatric hospital/	3816795
(adolesc* or babies or baby or boy? or boyhood or girlhood or child* or girl? or infan* or juvenil* or kid? or minor? or stepchild* or neonat* or neo-nat* or newborn* or new-born* or paediatric* or peadiatric* or pediatric* or pubert* or pubescen* or prepubert* or prepubescen* or preschool* or kindergarten* or school* or highschool* or youngster* or preteen* or teen* or toddler* or underage? or under-age? or youth*).ti,ab,kw	3804642
(pediatric* or paediatric* or infan* or child* or adolescen*).jn	330172
(pediatric* or paediatric* or infan* or child* or adolescen*).in	2010796
or/1-4	5885074
Diabetes Mellitus/ or exp insulin dependent diabetes mellitus/ or exp non insulin dependent diabetes mellitus/	962946
(diabet* or IDDM or NIDDM or T1DM or T2DM or T1D or T2D).ti,ab,kw	1081875
or/6-7	1275505
exp "physical activity, capacity and performance"/ or exp kinesiotherapy/ or dancing/ or fitness/ or exp sport/ or exp exercise test/ or exp ergometry/ or accelerometry/ or accelerometer/ or actimetry/ or exp oxygen consumption/ or exp sports equipment/	1735886
(exercis* or exertion or endurance or stamina or fitness* or sedentary or physiotherap* or physio therap* or kinesiotherap* or athletic* or workout or gym or gymnasi* or ymca* or work* out).ti,ab,kw	658806
((physical* or cardio* or aerobic*) adj4 (activ* or fit* or capacit* or effort* or inactiv* or conditioning or recondition* or train*)).ti,ab,kw	290584
((active or activit*) adj2 (living or play* or gaming or game* or lifestyle or behavio?r* or track* or monitor* or sensing or sensor* or detect*)).ti,ab,kw	112327
(crossfit* or HIIT or HIIE or plyometric* or personal train* or bodybuild* or body build* or weightlift* or (weight* adj2 lift*)).ti,ab,kw	10568

© 2024 Steiman De Visser H et al. *JAMA Network Open.*

((train* or condition* or program* or session*) adj4 (weight* or strength* or enduranc* or resistance or athletic* or fitness or aerobic* or interval* or flexibility or balance or muscle* or muscular or circuit or isometric* or isotonic* or agility or power)).ti,ab,kw	157049
(treadmill* or tread-mill* or walk* or stair* or jog* or swim* or running or runner* or run or runs or marathon* or triathl* or dance or dancing or climb* or cycling or bicycl* or bike or bikes or exercycl* or exerbik* or calisthenic* or skateboard* or rowing or rower or canoe* or kayak* or paddl* or skate or skating or ski or skiing or hike or hiking or jump or jumping or (skip* adj2 rope*)).ti,ab,kw	675233
(tai ji or tai chi or ai chi or yoga or pilates or stretch or stretches or stretching or flexibility or movement or qigong or qi gong or barre or tae bo or zumba or jazzercise).ti,ab,kw	515095
(pedomet* or accelerom* or actimet* or actigraph* or ergomet* or ergonomet* or navette? or shuttle or cycloergomet* or effort test* or andersen test* or maxim* oxygen* or peak oxygen* or oxygen consum* or oxygen require* or oxygen demand* or eurofit or prefit or pre-fit or president* challenge* or youth physical program* or physical best or stress test* or vo2max or vo2 max or an?erobic threshold* or metabolic equivalen* or work* rate* or work* capacit* or (time adj2 (exhaust* or fatigue*)) or ((step or steps) adj3 (count* or test* or daily or day or days or track*))).ti,ab,kw	229410
(exergam* or wii fit or zwift or peloton or kinect or fitbit*).ti,ab,kw	4628
(martial art* or boxing or kickboxing or karate or judo or jiu jitsu or jiujitsu or taekwondo or tae kwon do or fencing or wrestl* or aikido or fight* or grappl*).ti,ab,kw	57481
(sport* or golf* or aquatic* or gymnastic* or tennis or badminton or volleyball or ball or basketball or baseball or bowling or curls or curling or dodgeball or frisbee or lacrosse or racquet* or ringette or softball or polo or cricket or hockey or football or soccer or rugby or handball or mountaineer*).ti,ab,kw	255093
or/9-20	3190967
cross-sectional study/ or exp case control study/ or exp longitudinal study/ or prospective study/ or retrospective study/ or cohort analysis/ or observational study/ or comparative study/	3943404
(case control? or case comparison? or case base? or case referent or case referrent or case compeer or matched case?).tw,kw.	201555
(cohort adj (study or studies or analy$)).tw,kw.	406485
control* adj2 (before-after or "before and after").tw,kw	4958
historic* adj2 control*.tw,kw	15366

© 2024 Steiman De Visser H et al. *JAMA Network Open.*

((Follow up or followup) adj (study or studies)).tw,kw.	70619
((observation* or epidemiologic* or comparative or comparison or correlation*) adj2 (study or studies)).tw,kw.	580699
(longitudinal or retrospective or prospective).tw,kw.	2332244
(Cross section* or crosssection*).tw,kw.	637826
or/22-30	5274108
5 and 8 and 21 and 31	6155
(exp animal/ or nonhuman/) not exp human/	6815610
32 not 33	6037
limit 34 to (english language and yr="2000 -Current")	5657

© 2024 Steiman De Visser H et al. *JAMA Network Open.*

Cinahl

Search	Publications
(MH adolescence+) or (MH child+) or (MH "minors (legal)") or (MH puberty+) or (MH "adolescent health services") or (MH "child health services+") or (MH "adolescent medicine") or (MH pediatrics+) or (MH "adolescent health") or (MH "child health") or (MH pediatricians) or (MH neonatologists) or (MH "pediatric nurse practitioners+") or (MH "pediatric nursing+") or (MH "pediatric units") or (MH "hospitals, pediatric")	1100486
TI(adolesc* or babies or baby or boy or boys or boyhood or girlhood or child* or girl or girls or infan* or juvenil* or kid or kids or minor or minors or stepchild* or neonat* or neo-nat* or newborn* or new-born* or paediatric* or peadiatric* or pediatric* or pubert* or pubescen* or prepubert* or prepubescen* or preschool* or kindergarten* or school* or highschool* or youngster* or preteen* or teen* or toddler* or underage or underaged or "under age" or "under aged" or youth*) OR AB(adolesc* or babies or baby or boy or boys or boyhood or girlhood or child* or girl or girls or infan* or juvenil* or kid or kids or minor or minors or stepchild* or neonat* or neo-nat* or newborn* or new-born* or paediatric* or peadiatric* or pediatric* or pubert* or pubescen* or prepubert* or prepubescen* or preschool* or kindergarten* or school* or highschool* or youngster* or preteen* or teen* or toddler* or underage or underaged or "under age" or "under aged" or youth*)	1041847
SO(pediatric* or paediatric* or infan* or child* or adolescen*)	346521
AF(pediatric* or paediatric* or infan* or child* or adolescen*)	430744
S1 or S2 or S3 or S4	1615872
(MH "diabetes mellitus") or (MH "diabetes mellitus, type 1") or (MH "diabetes mellitus, type 2")	155938
TI(diabet* or IDDM or NIDDM or T1DM or T2DM or T1D or T2D) OR AB(diabet* or IDDM or NIDDM or T1DM or T2DM or T1D or T2D)	230332
S6 or S7	260027
(MH exercise+) or (MH exertion+) or (MH "physical activity") or (MH "physical fitness+") or (MH "therapeutic exercise+") or (MH dancing+) or (MH sports+) or (MH "exercise test+") or (MH ergometry) or (MH accelerometry+) or (MH "oxygen consumption+") or (MH accelerometers) or (MH "fitness trackers") or (MH pedometers) or (MH treadmills)	294548

© 2024 Steiman De Visser H et al. *JAMA Network Open.*

TI(exercis* or exertion or endurance or stamina or fitness* or sedentary or physiotherap* or (physio N1 therap*) or kinesiotherap* or athletic* or workout or gym or gymnasi* or ymca* or (work* N1 out)) OR AB(exercis* or exertion or endurance or stamina or fitness* or sedentary or physiotherap* or (physio N1 therap*) or kinesiotherap* or athletic* or workout or gym or gymnasi* or ymca* or (work* N1 out))	206749
TI((physical* or cardio* or aerobic*) N4 (activ* or fit* or capacit* or effort* or inactiv* or conditioning or recondition* or train*)) OR AB((physical* or cardio* or aerobic*) N4 (activ* or fit* or capacit* or effort* or inactiv* or conditioning or recondition* or train*))	102451
TI((active or activit*) N2 (living or play* or gaming or game* or lifestyle or behavior* or behaviour* or track* or monitor* or sensing or sensor* or detect*)) OR AB((active or activit*) N2 (living or play* or gaming or game* or lifestyle or behavior* or behaviour* or track* or monitor* or sensing or sensor* or detect*))	41706
TI(crossfit* or HIIT or HIIE or plyometric* or (personal N1 train*) or bodybuild* or (body N1 build*) or weightlift* or (weight* N2 lift*)) OR AB(crossfit* or HIIT or HIIE or plyometric* or (personal N1 train*) or bodybuild* or (body N1 build*) or weightlift* or (weight* N2 lift*))	5023
TI((train* or condition* or program* or session*) N4 (weight* or strength* or enduranc* or resistance or athletic* or fitness or aerobic* or interval* or flexibility or balance or muscle* or muscular or circuit or isometric* or isotonic* or agility or power)) OR AB((train* or condition* or program* or session*) N4 (weight* or strength* or enduranc* or resistance or athletic* or fitness or aerobic* or interval* or flexibility or balance or muscle* or muscular or circuit or isometric* or isotonic* or agility or power))	50081
TI(treadmill* or (tread N1 mill*) or walk* or stair* or jog* or swim* or running or runner* or run or runs or marathon* or triathl* or dance or dancing or climb* or cycling or bicycl* or bike or bikes or exercycl* or exerbik* or calisthenic* or skateboard* or rowing or rower or canoe* or kayak* or paddl* or skate or skating or ski or skiing or hike or hiking or jump or jumping or (skip* N2 rope*)) OR AB(treadmill* or (tread N1 mill*) or walk* or stair* or jog* or swim* or running or runner* or run or runs or marathon* or triathl* or dance or dancing or climb* or cycling or bicycl* or bike or bikes or exercycl* or exerbik* or calisthenic* or skateboard* or rowing or rower or canoe* or kayak* or paddl* or skate or skating or ski or skiing or hike or hiking or jump or jumping or (skip* N2 rope*))	134899

© 2024 Steiman De Visser H et al. *JAMA Network Open.*

TI("tai ji" or "tai chi" or "ai chi" or yoga or pilates or stretch or stretches or stretching or flexibility or movement or qigong or "qi gong" or barre or "tae bo" or zumba or jazzercise) OR AB("tai ji" or "tai chi" or "ai chi" or yoga or pilates or stretch or stretches or stretching or flexibility or movement or qigong or "qi gong" or barre or "tae bo" or zumba or jazzercise)	107341
TI(pedomet* or accelerom* or actimet* or actigraph* or ergomet* or ergonomet* or navette or shuttle or cycloergomet* or (effort N1 test*) or (andersen N1 test*) or (maxim* N1 oxygen*) or (peak N1 oxygen*) or (oxygen N1 consum*) or (oxygen N1 require*) or (oxygen N1 demand*) or eurofit or prefit or "pre fit" or (president* N1 challenge*) or ("youth physical" N1 program*) or "physical best" or (stress N1 test*) or vo2max or "vo2 max" or ((anerobic or anaerobic) N1 threshold*) or (metabolic N1 equivalen*) or (work* N1 rate*) or (work* N1 capacit*) or (time N2 (exhaust* or fatigue*)) or ((step or steps) N3 (count* or test* or daily or day or days or track*))) OR AB(pedomet* or accelerom* or actimet* or actigraph* or ergomet* or ergonomet* or navette or shuttle or cycloergomet* or (effort N1 test*) or (andersen N1 test*) or (maxim* N1 oxygen*) or (peak N1 oxygen*) or (oxygen N1 consum*) or (oxygen N1 require*) or (oxygen N1 demand*) or eurofit or prefit or "pre fit" or (president* N1 challenge*) or ("youth physical" N1 program*) or "physical best" or (stress N1 test*) or vo2max or "vo2 max" or ((anerobic or anaerobic) N1 threshold*) or (metabolic N1 equivalen*) or (work* N1 rate*) or (work* N1 capacit*) or (time N2 (exhaust* or fatigue*)) or ((step or steps) N3 (count* or test* or daily or day or days or track*)))	44732
TI(exergam* or "wii fit" or zwift or peloton or kinect or fitbit*) OR AB(exergam* or "wii fit" or zwift or peloton or kinect or fitbit*)	1592
TI((martial N1 art*) or boxing or kickboxing or karate or judo or "jiu jitsu" or jiujitsu or taekwondo or "tae kwon do" or fencing or wrestl* or aikido or fight* or grappl*) OR AB((martial N1 art*) or boxing or kickboxing or karate or judo or "jiu jitsu" or jiujitsu or taekwondo or "tae kwon do" or fencing or wrestl* or aikido or fight* or grappl*)	20736
TI(sport* or golf* or aquatic* or gymnastic* or tennis or badminton or volleyball or ball or basketball or baseball or bowling or curls or curling or dodgeball or frisbee or lacrosse or racquet* or ringette or softball or polo or cricket or hockey or football or soccer or rugby or handball or mountaineer*) OR AB(sport* or golf* or aquatic* or gymnastic* or tennis or	80552

© 2024 Steiman De Visser H et al. *JAMA Network Open.*

badminton or volleyball or ball or basketball or baseball or bowling or curls or curling or dodgeball or frisbee or lacrosse or racquet* or ringette or softball or polo or cricket or hockey or football or soccer or rugby or handball or mountaineer*)	
S9 or S10 or S11 or S12 or S13 or S14 or S15 or S16 or S17 or S18 or S19 or S20	643913
(MH "case control studies+") or (MH "correlational studies") or (MH "cross sectional studies") or (MH "prospective studies+") or (MH "controlled before-after studies") or (MH "historically controlled study") or (MH "nonexperimental studies") or (MH "retrospective design")	1025887
((case N1 control*) or (case N1 comparison*) or (case N1 base*) or "case referent" or "case referrent" or "case compeer" or (matched N1 case*))	116790
(cohort N1 (study or studies or analy*))	126336
control* N2 ("before-after" or "before and after")	1813
historic* N2 control*	2801
(("Follow up" or followup) N1 (study or studies))	17039
((observation* or epidemiologic* or comparative or comparison or correlation*) N2 (study or studies))	572463
(longitudinal or retrospective or prospective)	892262
((Cross N1 section*) or crosssection*)	276620
S22 or S23 or S24 or S25 or S26 or S27 or S28 or S29 or S30	1553509
S5 and S8 and S21 and S31	1943
(MH vertebrates+") not (MH human)	212257
S32 not S33	1933
S34 Limiters - Published Date: 20000101-; English Language	1794

© 2024 Steiman De Visser H et al. *JAMA Network Open.*

Sportdiscus

Search	Publications
DE "TEENAGERS" OR DE "OVERWEIGHT teenagers" OR DE "HIGH school students" or DE "CHILDREN" OR DE "BOYS" OR DE "CHILD development" OR DE "DANCE for children" OR DE "GIRLS" OR DE "OUTDOOR recreation for children" OR DE "OVERWEIGHT children" OR DE "SCHOOL children" OR DE "SELF-defense for children" or DE "YOUTH" OR DE "EXERCISE for youth" or DE "PUBERTY" OR DE "MENARCHE" or DE "ADOLESCENT health" or DE "CHILDREN'S health" OR DE "CHILD nutrition" OR DE "CHILDHOOD obesity" OR DE "PHYSICAL education for children" or DE "PEDIATRICS"	78940
TI(adolesc* or babies or baby or boy or boys or boyhood or girlhood or child* or girl or girls or infan* or juvenil* or kid or kids or minor or minors or stepchild* or neonat* or neo-nat* or newborn* or new-born* or paediatric* or peadiatric* or pediatric* or pubert* or pubescen* or prepubert* or prepubescen* or preschool* or kindergarten* or school* or highschool* or youngster* or preteen* or teen* or toddler* or underage or underaged or "under age" or "under aged" or youth*) OR AB(adolesc* or babies or baby or boy or boys or boyhood or girlhood or child* or girl or girls or infan* or juvenil* or kid or kids or minor or minors or stepchild* or neonat* or neo-nat* or newborn* or new-born* or paediatric* or peadiatric* or pediatric* or pubert* or pubescen* or prepubert* or prepubescen* or preschool* or kindergarten* or school* or highschool* or youngster* or preteen* or teen* or toddler* or underage or underaged or "under age" or "under aged" or youth*)	242071
S1 or S2	265183
DE "DIABETES" OR DE "DIABETES in children" OR DE "DIABETES in youth" OR DE "TYPE 1 diabetes" OR DE "TYPE 2 diabetes"	7130
TI(diabet* or IDDM or NIDDM or T1DM or T2DM or T1D or T2D) OR AB(diabet* or IDDM or NIDDM or T1DM or T2DM or T1D or T2D)	17878
S4 or S5	18402
DE "COHORT analysis"	1639
((case N1 control*) or (case N1 comparison*) or (case N1 base*) or "case referent" or "case referrent" or "case compeer" or (matched N1 case*))	7464
(cohort N1 (study or studies or analy*))	12446
control* N2 ("before-after" or "before and after")	297
historic* N2 control*	269

© 2024 Steiman De Visser H et al. *JAMA Network Open.*

(("Follow up" or followup) N1 (study or studies))	4891
((observation* or epidemiologic* or comparative or comparison or correlation*) N2 (study or studies))	60728
(longitudinal or retrospective or prospective)	65706
((Cross N1 section*) or crosssection*)	28802
S7 or S8 or S9 or S10 or S11 or S12 or S13 or S14 or S15	148835
S3 and S6 and S16	460
S17 Limiters - Published Date: 20000101-; Language: English	418

© 2024 Steiman De Visser H et al. *JAMA Network Open.*

eTable 2. Baseline Characteristics of Included Studies for Youth With Type 1 Diabetes and Cardiorespiratory Fitness

PMID	Author	year	country	n	Age (yrs)	Duration of Diabetes (yrs)	% Girls	n	Age	% girls
10749813	Riddell	2000	Canada	8	15.0 ± 1.0	n/a	0	6	15.0 ± 1.0	0
10647545	Riddell	2000	Canada	8	15.0 ± 2.0	6.7	n/a	8	15.0 ± 1.0	n/a
16109070	Komatsu	2005	Brazil	72	16.0 (9.0-20.0)	4.9	47	46	16.0 (10.0-18.0)	43
16299868	Heyman	2005	France	17	11.0 ± 0.3	1.0-9.0	0	18	10.0 ± 0.3	0
16489970	Galassetti	2006	USA	12	14.0 (12.0-16.0)	8.0	42	12	13.0 (12.0-16.0)	50
18446317	Gusso	2008	NZ	12	15.0 ± 0.4	6.1	100	20	15.0 ± 0.4	100
19411708	Rosa	2009	USA	16	14.0 ± 0.4	n/a	378	54	12.0 ± 1.0	52
20178512	Woo	2010	Korea	10	11.0 ± 1	3.9	0	10	12.0 ± 2.0	0
20411275	Maggio	2010	Switzerland	48	11.0 ± 0.4	3.3	n/a	85	10.0 ± 0.3	n/a
20826281	Trigona	2010	Switzerland	32	12.0 (10.0-13.0)	n/a	47	42	11.0 (10.0-12.0)	60
19915016	Nadeau	2010	USA	12	15.0 ± 3.0	7.5	50	12	16.0 ± 2.0	n/a
22803800	Cuenca-Garcia	2012	England	60	13.0 ± 2.0	5.0	33	37	12.0 ± 3.0	46
22353226	Lukacs	2012	Hungary	106	13.0 ± 2.0	5.1	50	130	13.0 ± 5.0	53
23935617	Jegdic	2013	Croatia	100	7.0-18.0	n/a	49	100	7.0-18.0	49
23965300	Faulkner	2014	USA	20	14.0 ± 2.0	5.8	45	10	15.0 ± 2.0	60
24444038	Nguyen	2014	Canada	16	14.0 ± 2.0	7.8	n/a	8	14.0 ± 3.0	n/a
25414156	Bjornstad	2015	USA	69	16.0 ± 2.0	6.3	n/a	13	15.0 ± 2.0	n/a
28050932	Abreu de Lima	2017	Brazil	45	12.0 ± 2.0	n/a	44	119	12.0 ± 1.0	47
n/a	Correa de Jesus	2019	Brazil	10	14.0 ± 2.0	n/a	44	12	13.0 ± 1.0	67
33712025	Wu	2021	China	48	14.0 ± 3.0	3.6	63	19	14.0 ± 3.0	58
33773936	Van Ryckeghem	2021	Belgium	19	15.0 ± 2.0	5.75	32	19	14.0 ± 1.0	26
33402367	Tommerdahl	2021	USA	195	15.0 ± 2.0	6.3	73	90	15.0 ± 2.0	66
34894721	Jahn	2022	USA	16	14.0 ± 0.5	6.8	24	14	14.0 ± 0.4	50

© 2024 Steiman De Visser H et al. *JAMA Network Open.*

eTable 3. Baseline Characteristics of Included Studies for Youth With Type 1 Diabetes and Physical Activity

				Cases				Controls		
PMID	Author	Year	Country	N	Age	% Girls	Duration of Diabetes	N	Age	% Girls
35270604	Elmesmari	2022	United Kingdom	20	7.4 ± 1.9	45.0	2.1 ± 1.4	20	7.3 ± 1.8	45.0
25092645	Mohammed	2014	Canada	66	16.0 ± 1.3	47.0	6.0 ± 3.9	54	16.3 ± 1.2	57.4
33712025	Wu	2021	China	48	14.0 ± 2.9	62.5	3.6 ± 2.3	19	13.6 ± 3.5	57.9
26197465	Mutlu	2015	Turkey	47	9.9 ± 1.6	55.3	2.8 ± 1.9	55	9.6 ± 1.6	49.1
33215796	Marshall	2021	South Wales	23	12.1 ± 2.1	60.9	5.0 ± 3.2	17	11.2 ± 2.2	35.3
33773936	Van Ryckeghem	2021	Belgium	19	14.8 ± 1.9	31.6	5.8 ± 3.0	19	14.4 ± 1.3	26.3
25191342	Bishop	2014	USA	300	15.3 ± 2.2	49.0	8.8 ± 0.1	100	14.9 ± 1.7	48.0
34018289	Cohen	2021	Israel	20	16.2 ± 2.5	60.0	10.1 ± 2.5	18	14.8 ± 2.3	61.1
34018289	Cohen	2021	Israel	20	14.1 ± 2	50.0	n/a	18	14.8 ± 2.3	61.1
27166715	Lukacs	2016	Finland	296	15.6 ± 1.9	0.0	7.0 ± 4.0	169	15.5 ± 1.6	0.0
27166715	Lukacs	2016	Finland	296	15.5 ± 1.8	44.9	7.3 ± 3.9	185	15.5 ± 1.6	100.0
25384048	Kummer	2014	Germany	629	15.3 ± 1.7	45.9	12.5 ± 1.6	613	14.6 ± 2.0	541.3
26758371	Caferolgu	2016	Turkey	70	13.0 (11.0-15.0)	45.7	3.5 (2.0-6.0)	72	12 (10.0-15.8)	50.0
18846317	Bener	2009	Qatar	170	10.5 ± 3.8	48.2	n/a	170	9.9 ± 4.2	52.9
32929477	Joseph	2020	USA	62	13.6 ± 1.7	100.0	4.8 ± 3.2	61	13.6 ± 1.9	100.0
22803800	Cuenca-Garcia	2012	England	60	12.5 ± 2.3	33.3	5.0 ± 3.7	37	12.0 ± 2.5	45.9
25092645	Mohammed	2014	Canada	66	16.0 ± 1.3	47.0	6.0	54	16.3 ± 1.2	57.4
26839891	Hensel	2016	Germany	40	11.5 ± 3.1	40.0	4.0 ± 3.5	44	11.4 ± 2.9	45.5
15686813	Moussa	2005	Kuwait	349	13.4 ± 3.2	62.2	n/a	409	13.4 ± 3.2	59.4
25414156	Bjornstad	2015	USA	69	15.5 ± 2.2	n/a	6.3 ± 3.8	13	15.1 ± 2.2	n/a
n/a	Mutlu	2017	Turkey	41	15.3 ± 1.6	56.1	n/a	38	14.7 ± 1.6	47.4
22353226	Lukacs	2012	Hungary	27	10.6 ± 1.5	100.0	4.9 ± 2.8	32	10.8 ± 1.2	100.0
22353226	Lukacs	2012	Hungary	26	15.8 ± 1.8	100.0	5.7 ± 2.8	37	16.0 ± 1.8	100.0
22353226	Lukacs	2012	Hungary	25	10.5 ± 1.5	0.0	3.8 ± 2.7	28	11.0 ± 1.1	0.0

© 2024 Steiman De Visser H et al. *JAMA Network Open.*

22353226	Lukacs	2012	Hungary	28	15.8 ± 1.8	0.0	6.1 ± 4.0	33	15.4 ± 1.7	0.0
20411275	Maggio	2010	Switzerland	48	10.7 ± 0.4	n/a	3.3 ± 0.3	85	10.1 ± 0.3	n/a
20826281	Trigona	2010	Switzerland	32	11.5 (10.2-12.8)	46.9	n/a	42	10.7 (9.6-11.8)	59.5
28050932	Abreu de Lima	2017	Brazil	45	12.4 ± 1.5	44.4	n/a	119	11.6 ± 0.7	42.9
17562573	Valerio	2017	Italy	138	13.6 ± 4.1	51.4	6.1 ± 3.8	269	12.9 ± 3.5	55.4
23196765	O'Neill	2012	USA	304	14.7 ± 3.2	50.7	n/a	127	14.5 ± 2.9	60.6
22480067	Fainardi	2011	Italy	129	12.1 ± 0.8	52.7	5.6 ± 2.8	214	12.2 ± 0.8	55.6
15975105	Sarnblad	2004	Sweden/UK	13	13.9 ± 1.3	100.0	4.7	22	13.8 ± 1.2	100.0
20457683	Lobelo	2010	USA	384	>10.0	51.0	3.8 ± 4.4	173	>10.0	59.0
23965300	Faulkner	2014	USA	20	14.2 ± 1.5	45.0	5.8 ± 2.9	10	14.6 ± 1.6	60.0
15941770	Massin	2005	Belgium	12	3.0 – 6.0	41.7	2.0 ± 1.3	59	3.0 – 6.0	52.5
15941770	Massin	2005	Belgium	52	7.0 – 12.0	61.5	4.6 ± 2.6	105	7.0 - 12.0	47.6
15941770	Massin	2005	Belgium	63	13.0 – 16.0	60.3	5.2 ± 3.6	36	13.0 – 16.0	52.8
16299868	Heyman	2005	France	17	10.5 ± 0.3	0.0	1.0 – 9.0	18	10.3 ± 0.3	0.0
22521039	Heyman	2012	France	19	15.9 ± 1.3	100.0	7.4 ± 4.5	19	16.6 ± 1.1	100.0
31546871	Czenczek-Lewandowska	2019	Poland	215	12.6 ± 3.3	60	3.9 ± 3.3	115	n/a	n/a
34924277	Francisco dos Santos Haber	2021	Brazil	32	13.6 ± 3.0	n/a	5.5 ± 3.2	30	151.3 mo. ± 46.4	n/a
34924277	Francisco dos Santos Haber	2021	Brazil	30	13.9 ± 4.1	n/a	5.6 ± 5.1	30	151.3 mo. ± 46.4	n/a
19915016	Nadeau	2010	USA	12	14.8 ± 2.6	50	7.5 ± 4.0	12	15.6 ± 1.8	0.0
24444038	Nguyen	2014	Canada	8	13.8 ± 3	n/a	n/a	8	13.6 ± 2.6	0.0
24444038	Nguyen	2014	Canada	8	14.2 ± 1.3	n/a	n/a	8	13.6 ± 2.6	0.0

© 2024 Steiman De Visser H et al. *JAMA Network Open.*

eTable 4. Baseline Characteristics of Included Studies for Youth With Type 2 Diabetes and Physical Activity

				Cases				Controls		
PMID	Author	Year	Country	N	Age	% Girls	Duration of Diabetes	N	Age	% Girls
26994128	Bjornstad	2016	USA	37	15.4±2.3	70.3	4.6 (1.5-8.0)	41	14.4±2	70.7
26994128	Bjornstad	2016	USA	37	15.4±2.3	70.3	4.6 (1.5-8.0)	33	14.9±2.1	54.5
18503498	Shaibi	2008	USA	13	16.4±0.6	0.0	2.4±1.8	13	15.2±0.5	0.0
18945923	West	2009	USA	106	15.7±4.4	68.9	1.5±1.4	189	14.3±4.6	59.8
33776940	Miranda-Lora	2021	USA	97	12.9±2.6	46.4	n/a	83	12.5±2.9	45.8
22222927	Wittmeier	2012	Canada	27	15±1.5	59.3	n/a	97	15±1.7	69.1
22222927	Wittmeier	2012	Canada	27	15±1.5	59.3	n/a	13	16±1.8	61.5
23196765	O'Neill	2012	USA	49	15.6±2.9	75.5	1.4±1.4	127	14.5±2.9	60.6
20457683	lobelo	2010	USA	90	10<	70.0	1.7±1.6	173	10<	59.0
23965300	Faulkner	2014	USA	9	14.7±1.8	88.9	1.6±1.4	10	14.6±1.6	60.0
19584191	Nadeau	2009	USA	14	14.6±2.2	50.0	1.6±1.6	12	15.6±1.8	50.0

© 2024 Steiman De Visser H et al. *JAMA Network Open.*

eTable 5. Baseline Characteristics of Included Studies for Youth With Type 2 Diabetes and Cardiorespiratory Fitness

				Cases				Controls		
**PMID**	**Author**	**Year**	**Country**	**N**	**Age**	**% Girls**	**Duration of Diabetes**	**N**	**Age**	**% Girls**
**25387459**	Yardley	2015	Canada	23	15.3 ± 1.6	43.5	n/a	15	16.2±2	66.7
**25387459**	Yardley	2015	Canada	23	15.3±1.6	43.5	n/a	23	15.1±1.5	43.5
**26994128**	Bjornstad	2016	USA	37	15.4±2.3	70.3	4.6 (1.5-8.0)	41	14.4±2	70.7
**26994128**	Bjornstad	2016	USA	37	15.4±2.3	70.3	4.6 (1.5-8.0)	33	14.9±2.1	54.5
**18503498**	Shaibi	2008	USA	13	16.4±0.6	0.0	2.4 ± 1.8	13	15.2±0.5	0.0
**28626942**	Hodges	2018	USA	12	16±2.1	50.0	n/a	22	16.2±2.3	45.5
**28626942**	Hodges	2018	USA	12	16±2.1	50.0	n/a	10	15.8±2.1	40.0
**33776940**	Miranda-Lora	2021	USA	97	12.9±2.6	46.4	n/a	83	12.5±2.9	45.8
**22222927**	Wittmeier	2012	Canada	27	15±1.5	59.3	n/a	97	15±1.7	69.1
**22222927**	Wittmeier	2012	Canada	27	15±1.5	59.3	n/a	13	16±1.8	61.5
**33402367**	Tommerdahl	2021	USA	59	15.4±2.3	71.2	2.1 ± 1.9	43	15±2.1	62.8
**33402367**	Tommerdahl	2021	USA	59	15.4±2.3	71.2	2.1 ± 1.9	47	14.5±2	68.1
**18446317**	Gusso	2008	New Zealand	8	14.9±0.5	100.0	2.5 ± 1	10	15.2±0.4	100.0
**18446317**	Gusso	2008	New Zealand	8	14.9±0.5	100.0	2.5 ± 1	10	15.1±0.4	100.0
**23965300**	Faulkner	2014	USA	9	14.7±1.8	88.9	1.6 ± 1.4	10	14.6±1.6	60.0
19584191	Nadeau	2009	USA	14	14.6±2.2	50.0	1.6 ± 1.6	12	15.6±1.8	50.0

© 2024 Steiman De Visser H et al. *JAMA Network Open.*

eTable 6. Ethnicity of Participants in Each Study of Youth With Type 2 Diabetes Within Studies That Reported It

	Cardiorespiratory fitness		Physical Activity	
**Ethnicity**	**Cases**	**Controls**	**Cases**	**Controls**
Indigenous	26.2%	10.7%	8.2%	3.9%
Black	6.2%	0.4%	50.0%	23.5%
Hispanic	37.9%	18.1%	17.2%	14.5%
White	10.3%	45.0%	23.4%	55.0%
Other	19.3%	25.8%	1.3%	2.8%

eTable 7. Ethnicity of Participants in Each Study of Youth With Type 1 Diabetes Within Studies That Reported It

	Cardiorespiratory fitness		Physical Activity	
**Ethnicity**	**Cases**	**Controls**	**Cases**	**Controls**
Indigenous	0	0	0	0
Black	7.82	19.5	0.9	0.8
Hispanic	5.21	5.9	7.5	14.4
white	75.8	61.7	84.4	75.3
Other	11.2	12.9	7.2	9.5

© 2024 Steiman De Visser H et al. *JAMA Network Open.*

eFigure 1. Flow Chart Describing Search and Screening Results for Studies the Were Included in the Meta-Analysis

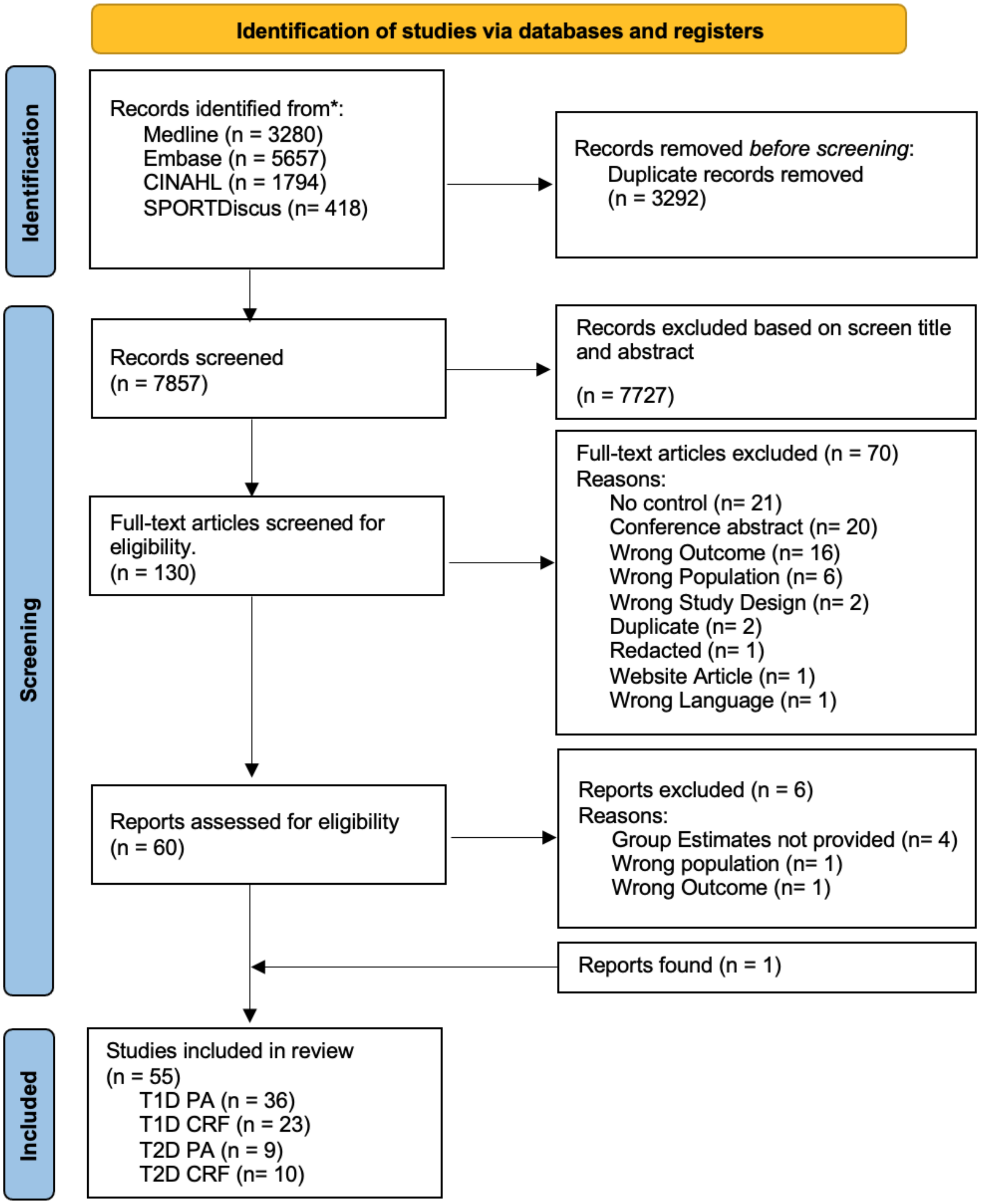

© 2024 Steiman De Visser H et al. *JAMA Network Open.*

eFigure 2. Differences in Objectively Measured Physical Activity Between Youth With Type 2 Diabetes and Controls

Study	Experimental Mean	Experimental SD	Experimental Total	Control Mean	Control SD	Control Total	Weight	Std. Mean Difference IV, Random, 95% CI
Wittmeier	4475.00	2382.0000	27	7269.46	2916.9200	110	37.6%	−0.98 [−1.42; −0.55]
O'Neill	4959.00	3474.0000	49	6870.00	3521.0000	127	50.2%	−0.54 [−0.88; −0.21]
Faulkner	9.10	7.8000	9	17.10	18.2000	10	12.2%	−0.53 [−1.46; 0.39]
**Total (95% CI)**			**85**			**247**	**100.0%**	**−0.71 [−1.36; −0.05]**

Heterogeneity: $Tau^2 = 0.0321$; $Chi^2 = 2.60$, df = 2 (P = 0.27); $I^2 = 23\%$

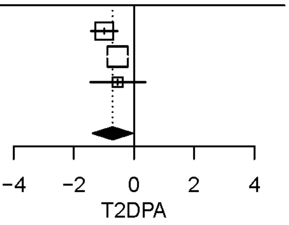

eFigure 3. Differences in Objectively Measured Physical Activity Between Youth With Type 1 Diabetes and Controls

Study	Experimental Mean	Experimental SD	Experimental Total	Control Mean	Control SD	Control Total	Weight	Std. Mean Difference IV, Random, 95% CI
Maggio	497.00	54.0000	48	668.00	35.0000	85	7.7%	−3.97 [−4.57; −3.37]
Elmesmari_2	8201.00	3231.0000	20	10959.00	2024.0000	20	7.5%	−1.00 [−1.66; −0.34]
Elmesmari_1	1.70	0.7000	20	2.20	0.4000	20	7.6%	−0.86 [−1.51; −0.21]
Wu	2.09	0.4100	48	2.41	0.6000	19	7.9%	−0.67 [−1.22; −0.13]
Faulkner	8.90	10.0000	20	17.10	18.2000	10	7.2%	−0.60 [−1.38; 0.17]
Marshall	82.90	37.2000	23	114.00	72.1000	17	7.6%	−0.56 [−1.20; 0.08]
sarnblad	505.00	145.0000	13	572.00	125.0000	22	7.4%	−0.49 [−1.19; 0.20]
Trigona	567.10	313.1473	32	694.90	291.9640	42	8.1%	−0.42 [−0.88; 0.05]
Nguyen	45.90	16.3000	8	53.80	28.1000	8	6.5%	−0.33 [−1.31; 0.66]
Czenczek−Lewandowska	254.80	158.7000	215	268.30	83.7000	115	8.5%	−0.10 [−0.32; 0.13]
Nadeau	64.00	7.0000	12	64.00	11.0000	12	7.1%	0.00 [−0.80; 0.80]
Cuenca−Garcia	26995.10	11668.6000	60	26477.10	10157.6000	37	8.2%	0.05 [−0.36; 0.46]
O'Neill	7413.00	3415.0000	304	6870.00	3521.0000	127	8.6%	0.16 [−0.05; 0.36]
**Total (95% CI)**			**823**			**534**	**100.0%**	**−0.67 [−1.17; −0.17]**

Heterogeneity: $Tau^2 = 0.7424$; $Chi^2 = 179.77$, df = 12 (P < 0.01); $I^2 = 93\%$

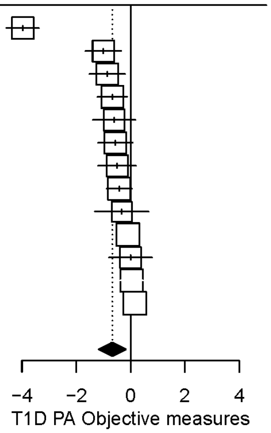

© 2024 Steiman De Visser H et al. *JAMA Network Open.*

eFigure 4. Funnel Plot Demonstrating No Publication Bias in Studies Examining Differences in Cardiorespiratory Fitness Between Youth With Type 2 Diabetes and Controls

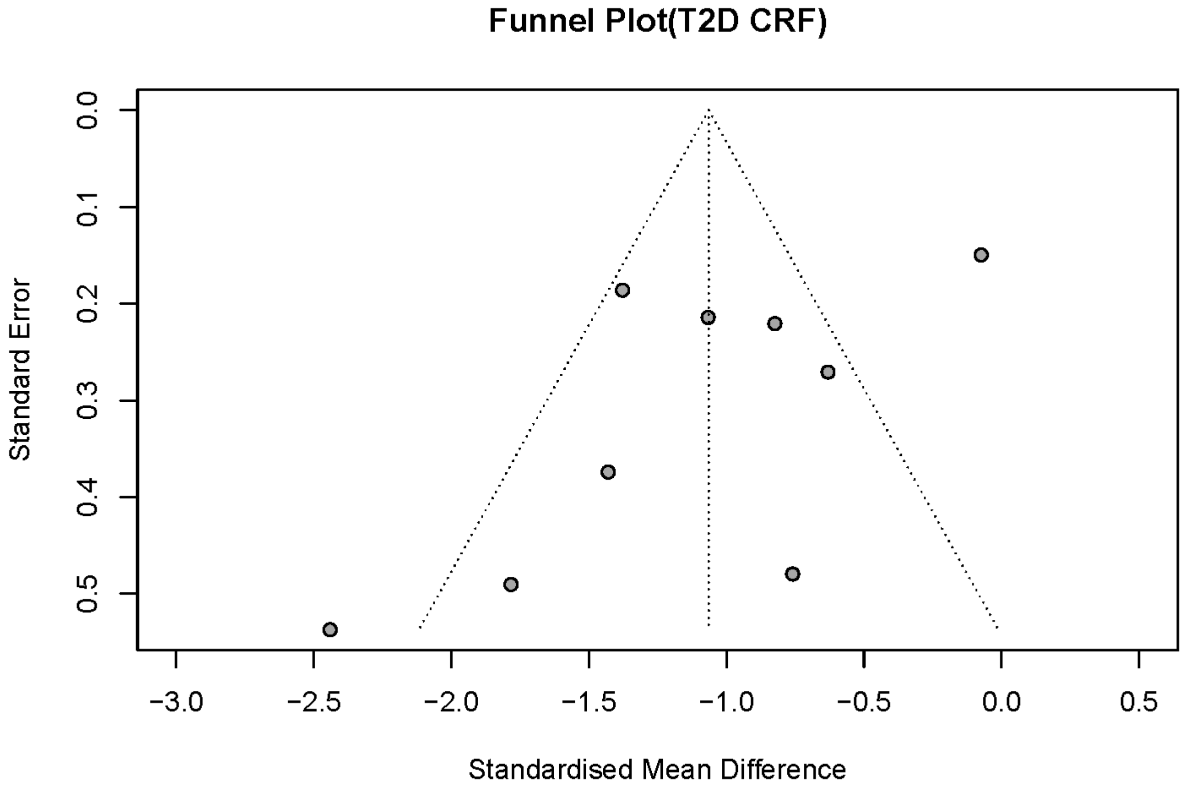

© 2024 Steiman De Visser H et al. *JAMA Network Open.*

eFigure 5. Funnel Plot Demonstrating No Publication Bias in Studies Examining Differences in Cardiorespiratory Fitness Between Youth With Type 1 Diabetes and Controls

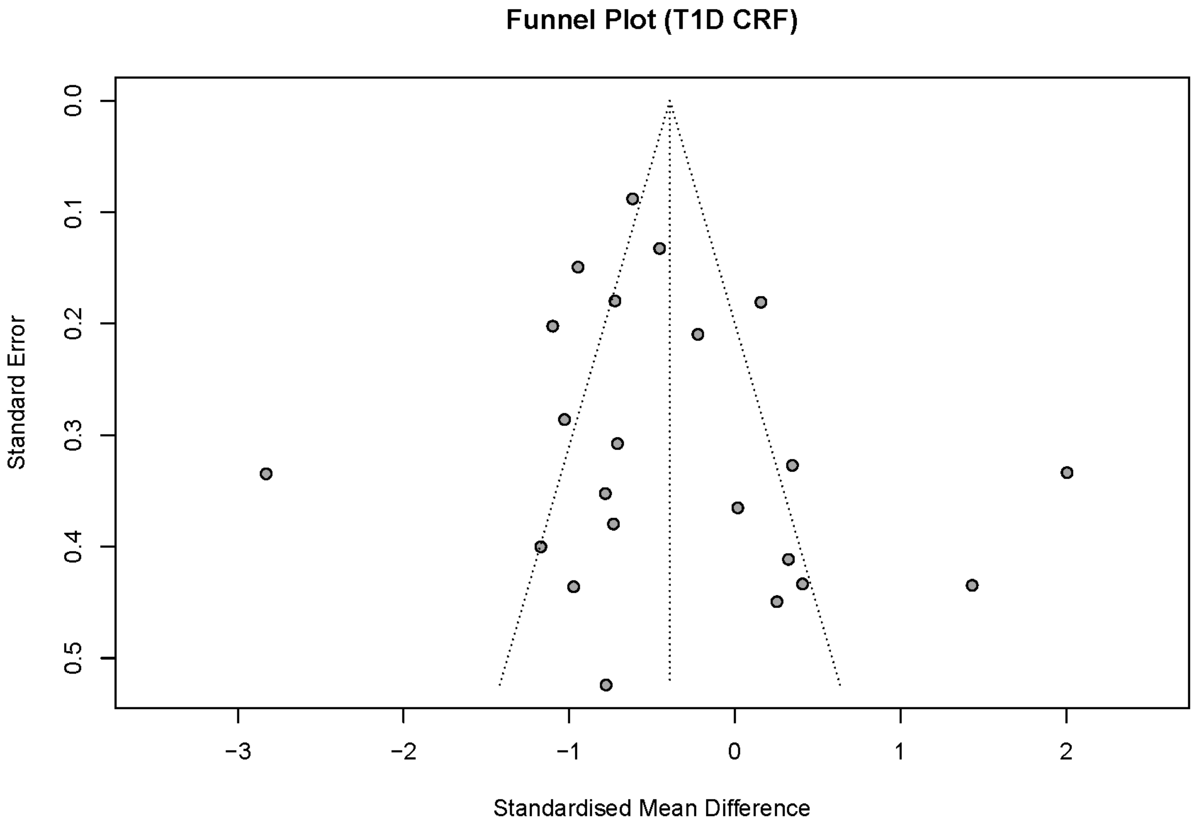

© 2024 Steiman De Visser H et al. *JAMA Network Open.*

eFigure 6. Funnel Plot Demonstrating No Publication Bias in Studies Examining Differences in Physical Activity Between Youth With Type 2 Diabetes and Controls

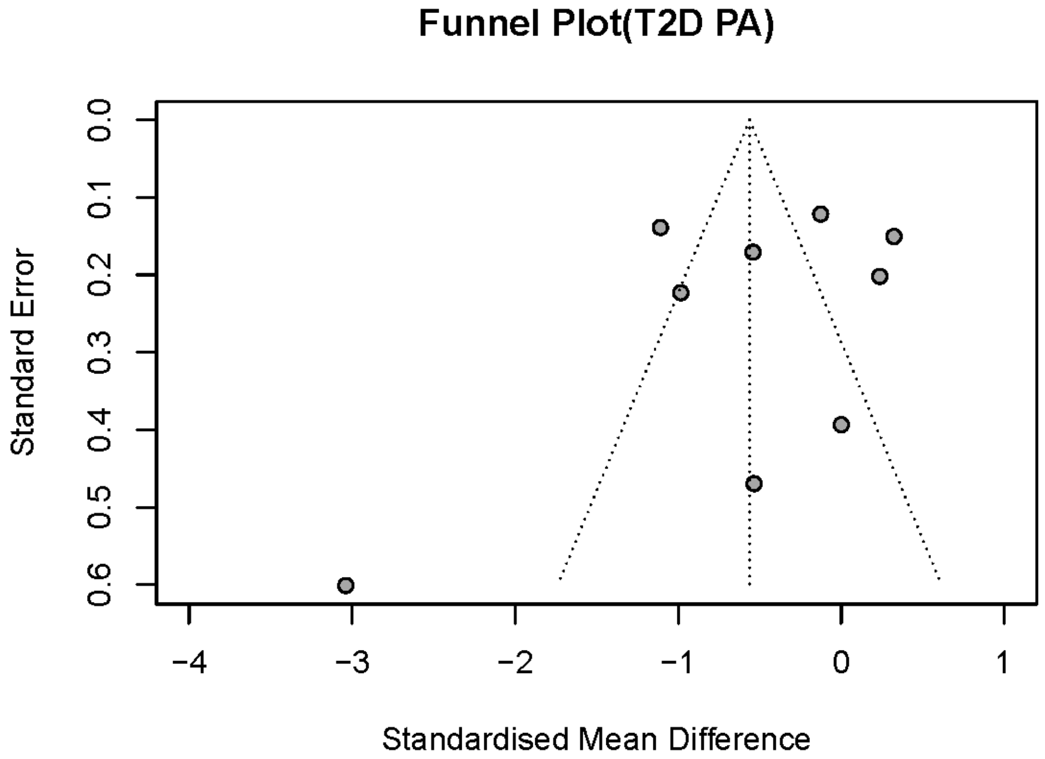

© 2024 Steiman De Visser H et al. *JAMA Network Open.*

eFigure 7. Funnel Plot Demonstrating No Publication Bias in Studies Examining Differences in Physical Activity Between Youth With Type 1 Diabetes and Controls

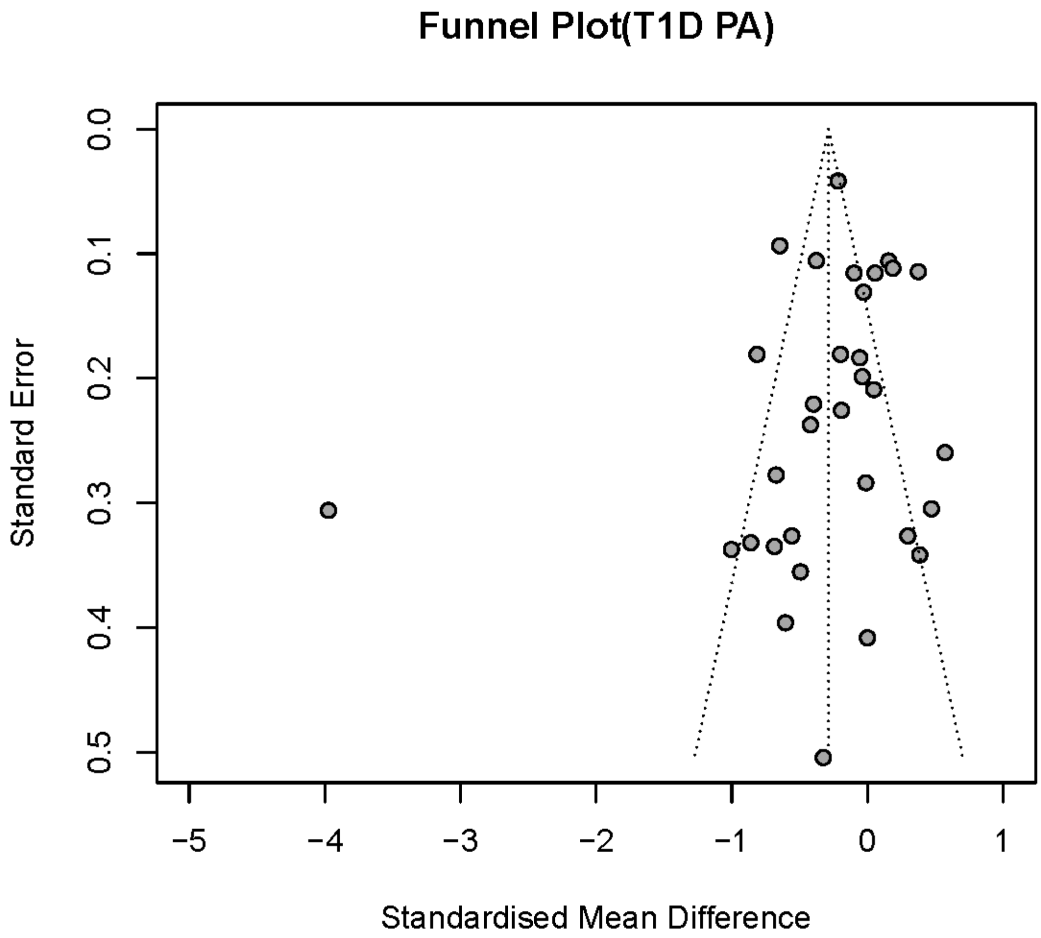

© 2024 Steiman De Visser H et al. *JAMA Network Open.*
