# Supplementary material for: Cardiorespiratory Fitness and Physical Activity in Pediatric Diabetes: A Systemic Review and Meta-Analysis
Source: JAMA Netw Open. 2024 Feb 23;7(2):e240235. doi: 10.1001/jamanetworkopen.2024.0235 (PMC10891480; doi:10.1001/jamanetworkopen.2024.0235)
Supplement: Supplement 2. — Data Sharing Statement [file jamanetwopen-e240235-s002.pdf]

Data Sharing Statement

Steiman De Visser. Cardiorespiratory Fitness and Physical Activity in Pediatric Diabetes. *JAMA Netw Open*. Published February 23, 2024. doi:10.1001/jamanetworkopen.2024.0235

Data
Data available: Yes
Data types: Data (not involving human participants)
How to access data: We can provide data that was extracted from the studies included in the meta-analysis
When available: With publication

Supporting Documents
Document types: None

Additional Information
Who can access the data: Anyone requesting data.
Types of analyses: For any specified purpose.
Mechanisms of data availability: With a signed data access agreement
Any additional restrictions: None
